# Capture of toxic gases in MOFs: SO_2_, H_2_S, NH_3_ and NO_*x*_

**DOI:** 10.1039/d1sc01609a

**Published:** 2021-04-28

**Authors:** Eva Martínez-Ahumada, Mariana L. Díaz-Ramírez, Miriam de J. Velásquez-Hernández, Vojtech Jancik, Ilich A. Ibarra

**Affiliations:** Laboratorio de Fisicoquímica y Reactividad de Superficies (LaFReS), Instituto de Investigaciones en Materiales, Universidad Nacional Autónoma de México Circuito Exterior s/n, CU, Del. Coyoacán, 04510 Ciudad de México Mexico argel@unam.mx +52(55) 5622-4595; Department of Chemistry, Mississippi State University Box 9573 Mississippi 39762 USA; Institute of Physical and Theoretical Chemistry, Graz University of Technology 8010 Graz Austria; Universidad Nacional Autónoma de México, Instituto de Química, Ciudad Universitaria Ciudad de México Mexico vjancik@unam.mx; Centro Conjunto de Investigación en Química Sustentable UAEM-UNAM Carr. Toluca-Atlacomulco Km 14.5 Toluca Estado de México 50200 Mexico

## Abstract

MOFs are promising candidates for the capture of toxic gases since their adsorption properties can be tuned as a function of the topology and chemical composition of the pores. Although the main drawback of MOFs is their vulnerability to these highly corrosive gases which can compromise their chemical stability, remarkable examples have demonstrated high chemical stability to SO_2_, H_2_S, NH_3_ and NO_*x*_. Understanding the role of different chemical functionalities, within the pores of MOFs, is the key for accomplishing superior captures of these toxic gases. Thus, the interactions of such functional groups (coordinatively unsaturated metal sites, μ-OH groups, defective sites and halogen groups) with these toxic molecules, not only determines the capture properties of MOFs, but also can provide a guideline for the desigh of new multi-functionalised MOF materials. Thus, this perspective aims to provide valuable information on the significant progress on this environmental-remediation field, which could inspire more investigators to provide more and novel research on such challenging task.

## Abstract 2

MOFs are promising candidates for the capture of toxic gases such as SO_2_, H_2_S, NH_3_ and NO*x*. Understanding the role of different chemical functionalities, within the pores of MOFs, is the key for accomplishing superior captures of these toxic gases.

## Graphical abstract

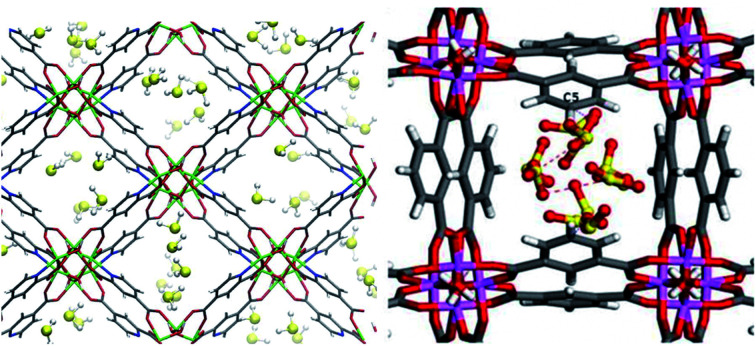

## 1. Introduction

The accelerated growth of our modern society demands huge amounts of energy. Unwittingly, in order to provide these high energetic levels, an indiscriminate combustion of large volumes of fossil fuels occurs, leading to an incommensurable release of toxic pollutants to the atmosphere. Emissions of anthropogenic air pollutants generate a vast range of health complications (*e.g.*, premature death and morbidity). Additionally, these air pollutants are also responsible for the reduction of the biodiversity, crop damages and acidification of soils and waters.^1^ As an example of the adverse impacts to humans, the World Health Organization (WHO) recently announced that air pollution was responsible for the premature mortality of approximately 4.2 million people in 2016 alone.^2^ In fact, air pollution is now the single largest environmental health risk worldwide since it is responsible for one in eight premature global deaths.^3^ For example, PM_2.5_ (fine inhalable particles, with diameters that are generally 2.5 micrometres and smaller) are responsible for approximately half of the deaths related to air pollution.^4^ Thus, PM_2.5_ have been classified as the air pollutant with the highest impact in premature mortality,^5^ and a reduction of their emissions is crucial. Such reduction can be achieved in two steps: (i) mitigation of primary PM_2.5_ emissions and (ii) extenuation of secondary inorganic aerosols (SIA); which are oxidised from precursor emissions such as sulphur dioxide (SO_2_), nitrogen oxides (NO_*x*_), ammonia (NH_3_) and volatile organic compounds (VOCs).^6^ Thus, in order to extenuate these emissions, different actions have been taken, such as the shutdown of some coal-fired power plants and their replacement by thermoelectric power plants, importing electricity from other countries with strict restrictions on the electricity production and the use of more environmentally friendly energy sources.^7^ In addition to these actions, the development of efficient technologies for the capture of toxic gases (*e.g.*, NO_*x*_, SO_2_, NH_3_ and H_2_S) from static and mobile sources is necessary, in order to achieve a cleaner environment.^8^

Porous metal–organic frameworks (MOFs) or porous coordination polymers (PCPs) are amongst the most promising candidates for the capture of these toxic gases since their sorption selectivity is directly tuneable as a function of the topology and chemical composition of the pores.^9^ Undoubtedly, for NO_*x*_, SO_2_, NH_3_ and H_2_S capture, there is a substantial emphasis on optimising the interactions between MOF materials and these toxic molecules, leading to the discovery of new functional porous materials with enhanced gas adsorption properties.^10^ Although MOF materials have shown very promising capabilities for the capture of these toxic gases, their main drawback is their vulnerability to these highly corrosive molecules capable of compromising the chemical stability of the MOFs. NO_*x*_, SO_2_, NH_3_ and H_2_S can disrupt the coordination bonds between the organic ligands (*e.g.*, carboxylate) and the metal centres, occasioning the breakdown of the MOF structure. Therefore, chemical stability of the MOFs is a fundamental requirement for the capture of these toxic gases. The present contribution aims to provide a useful reference in the field of capturing toxic gases in MOFs, emphasising on their chemical stability and the role of the functional groups to enhance such captures. We hope to encourage more research groups to explore the exciting frontiers of science in environmental-remediation applications for MOFs.

## 2. Sulphur dioxide

The anthropogenic release of SO_2_ into the atmosphere is mainly due to the combustion of fossil fuels (*e.g.*, electric power generation). One of the first environmental problems related to SO_2_ was observed during the last century in the acidic deposition.^11^ To avoid this, since 1990 many European countries have considerably reduced their SO_2_ emissions.^12^ Nevertheless, the rapid industrialisation of some developing countries has caused a constant presence of SO_2_ in the atmosphere,^13^ risking the health of millions of people (*vide supra*).

SO_2_ is a colourless, non-flammable and corrosive gas with high solubility (120 g l^−1^) in water. Among the technologies for SO_2_ capture, wet and dry flue-gas desulfurization (FGD) processes are commonly used,^14^ where the cost and recyclability depend on the used technology. Among them, alkaline solutions, activated carbons, zeolites and silica have typically showed low SO_2_ adsorption capacities, accompanied by the corrosion of pipelines and high energy regeneration overheads.^15^ Thus, the design of new materials is extremely necessary in order to meet all the challenges that the capture of SO_2_ signifies. MOF materials are an exciting alternative for the capture of SO_2_ as they have demonstrated promising results for the SO_2_ capture assignment, mainly due to their specific chemical functionality and pore dimensions.^16^ The following section focuses on the highlights of remarkable MOF materials stable towards SO_2_, with the emphasis not only on their capture performance, but also on showing the role of different chemical functionalities which are the key for the SO_2_ capture.

### 2.1 MOFs for SO_2_ capture

The chemical nature of the SO_2_ molecule and its main binding modes: oxygen, an electron-rich atom that can act as a Lewis base; and sulphur that can be a Lewis acid site, have been exploited to improve its adsorption on the porous surface of the MOFs. Due to the specific functionalisation of these MOFs, interesting SO_2_ capture results have been achieved. Such functionalisation can be classified in four main categories: (i) coordinatively unsaturated metal sites (open metal sites), (ii) μ-OH groups, (iii) defective sites and (iv) halogen functionalisation.

#### (i) Coordinatively unsaturated metal sites

**Fig. 1:**
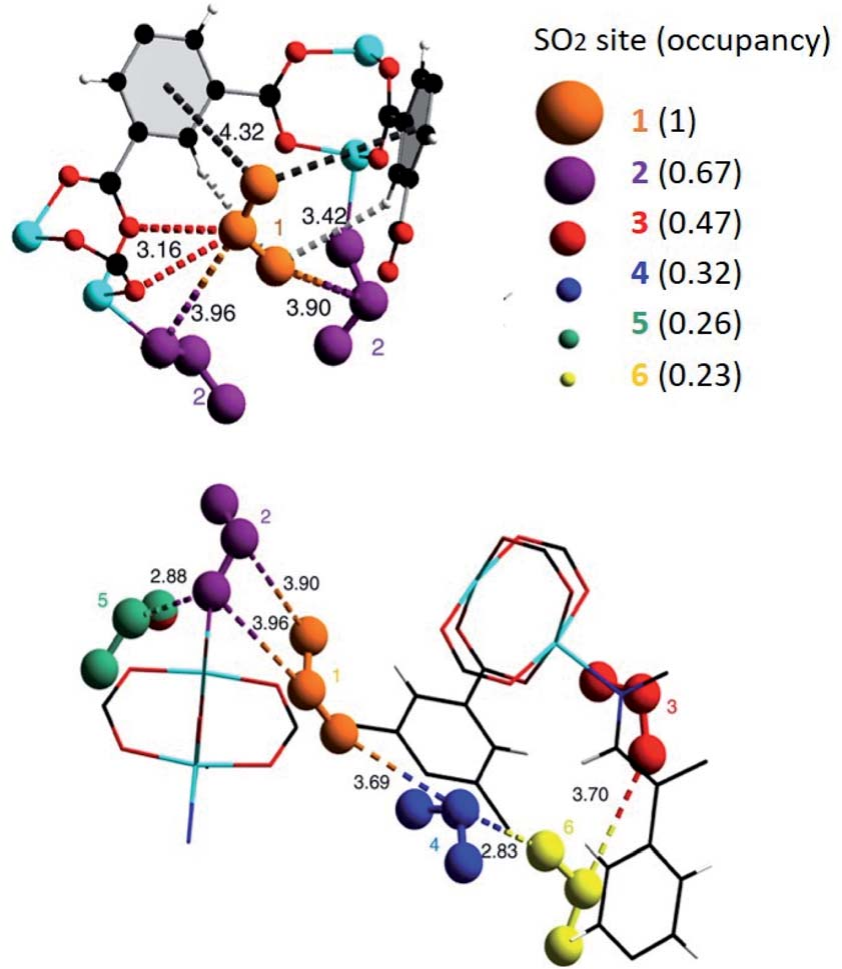
SO_2_ positions within MFM-170 pores obtained from *in situ* single-crystal X-ray diffraction. Purple SO_2_ molecule is located at the thermodynamically strongest binding site. Cu, cyan; C, black; O, red; N, blue. Reprinted (adapted) with permission from ref. 19. Copyright (2019) Springer Nature.

Although some research has proven the capture of SO_2_ by different MOFs, the majority of these examples have shown to be chemically unstable upon SO_2_ exposure.^17^ For example, MOF-177 (an open Zn^2+^ sites system reported by Janiak and co-workers^18^) holds the SO_2_ capture record (25.7 mmol g^−1^ at 298 K and 1 bar) but suffers a partial structural degradation, after the exposure to SO_2_, as demonstrated by PXRD and a significant BET surface area reduction.^18^ Conversely, MFM-170 an open Cu^2+^ sites MOF; [Cu_2_(L)] (H_4_L = 4′,4′′′-(pyridine-3,5-diyl)bis([1,1′-biphenyl]-3,5-dicarboxylic acid)), not only demonstrated to be highly stable to SO_2_ (17.5 mmol g^−1^ at 298 K and 1 bar), but also to humid SO_2_.^19^ Yang and Schröder elegantly demonstrated the reversible coordination of SO_2_ to open Cu^2+^ sites in MFM-170 by *in situ* synchrotron single-crystal X-ray diffraction, *in situ* FTIR spectroscopy and *in situ* inelastic neutron scattering (INS).^19^ In brief, all of this comprehensive experimental evidence corroborated that the Cu^2+^ site is the thermodynamically strongest SO_2_ binding site (see Fig. 1), but is sufficiently weak to be almost entirely desorbed upon reducing the pressure. The material can also be fully regenerated by heating to 400 K without any loss of crystallinity even after completing fifty SO_2_ adsorption–desorption cycles at 298 K.^19^

Our group reported a partially fluorinated version of MIL-101(Cr) named MIL-101(Cr)-4F(1%).^20^ The particularity of MIL-101(Cr)-4F(1%) is the different chemical character of the Cr^3+^ metal centres, in comparison to MIL-101(Cr). The incorporation of fluorine in MIL-101(Cr) promoted a higher acidity of some of the Cr^3+^ metal centres (open metal sites), due to the capability of fluorine to attract electrons.^20^ Such difference afforded a total SO_2_ capture of 18.4 mmol g^−1^ at 298 K and up to 1 bar, chemical stability towards dry and humid SO_2_ and an exceptional cycling performance with facile regeneration.^20^

This uptake represents the highest SO_2_ capture for a structurally stable MOF material. In addition, *in situ* DRIFT spectroscopy upon the adsorption of CO demonstrated the efficient packing of SO_2_ molecules within MIL-101(Cr)-4F(1%). Thus, the SO_2_ uptake mechanism takes place in three stages: (i) adsorption at acid (Lewis) Cr^3+^ sites with a relatively high heat of adsorption for SO_2_; (ii) adsorption at both acid (Lewis and Brønsted) sites of MIL-101(Cr)-4F(1%) and (iii) adsorption within the cavities of this MOF material. Up to this point, we have presented three examples (MOF-177, MFM-170 and MIL-101(Cr)-4F(1%)) of MOF materials with coordinatively unsaturated metal sites that demonstrated the highest SO_2_ captures for any MOF. One material (MOF-177) demonstrated poor structural stability towards SO_2_*versus* the high structural stability of MFM-170 and MIL-101(Cr)-4F(1%) in the presence of SO_2_ and humid SO_2_. Unwittingly, the fundamental question arises: why is MOF-177 unstable while MFM-170 and MIL-101(Cr)-4F(1%) are stable to SO_2_? In order to answer this question, their comparison is presented in Table 1, allowing to propose a stability hypothesis for these materials.

**Table 1:** Comparison of structural characteristics in metal–organic frameworks with the highest SO_2_ capture

Material	MOF-177	MFM-170	MIL-101(Cr)-4F(1%)
Ligand	H_3_BTB	H_4_L = 4′,4′′′-(pyridine-3,5-diyl)bis([1,1′-biphenyl]-3,5-dicarboxylic acid)	
			H_2_BDC/H_2_BDC-4F
SBU	[Zn_4_(μ_4_-O)(O_2_CR)_6_]	[Cu_2_(O_2_CR)_4_]	[Cr_3_(μ_3_-O)(O_2_CR)_6_]
	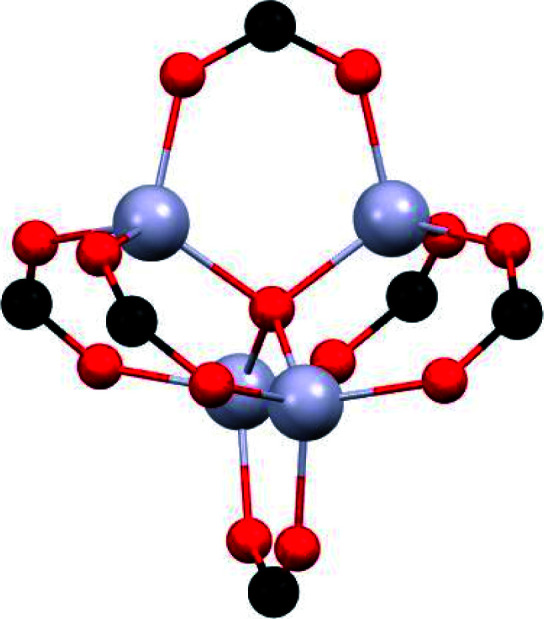	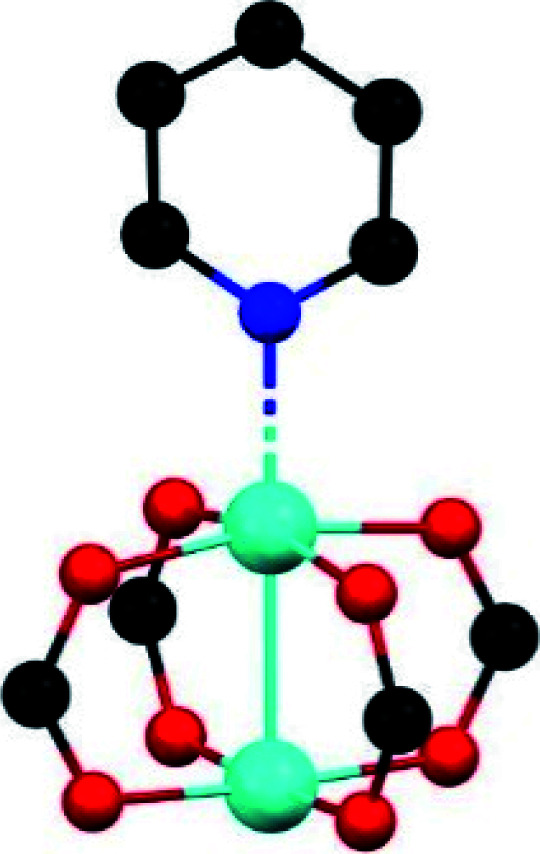	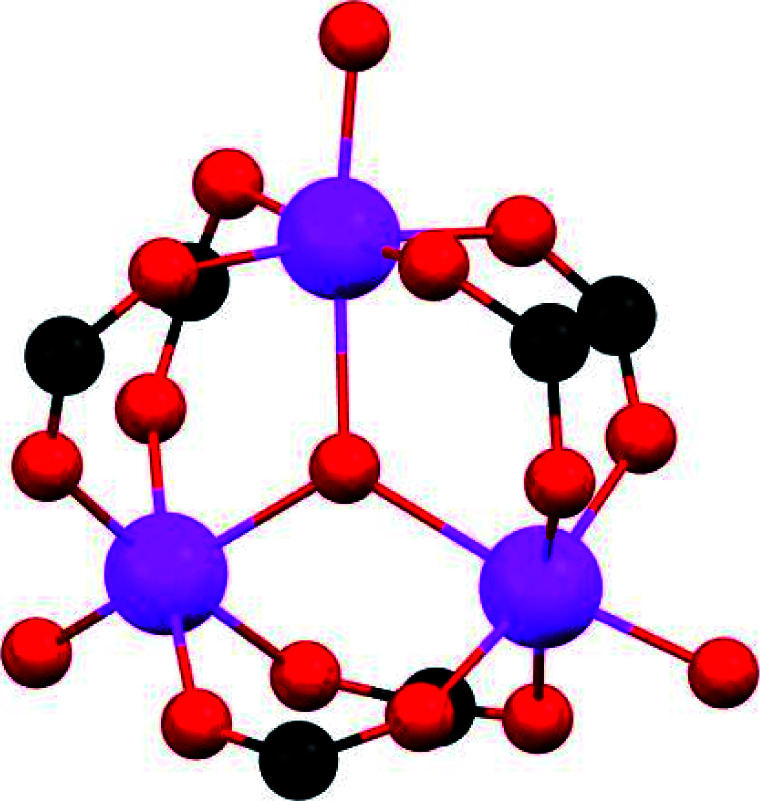
BET surface area [m^2^ g^−1^]	4100	2408	2176
Pore volume [cm^3^ g^−1^]	1.51	0.88	1.19
SO_2_ adsorption [mmol g^−1^]	25.7	17.5	18.4
Packing density [cm^3^ g^−1^]	1.09	1.27	1.00
Stability under SO_2_	Partial destruction after SO_2_ exposure	Stable after 50 cycles	Stable after 50 cycles

The design of stable frameworks for capture, confinement and release of corrosive gases uses tools centred in the robustness of the metal-linker bond, the use of inert or higher-valent metals and, the use of poly-nuclear secondary building units (SBUs).^21^ In the case of MOF-177, MFM-170 and MIL-101(Cr)-4F(1%), these exhibit at least one of such design tools. These three MOFs are composed of carboxylate ligands and robust SBUs, this multinuclearity gives, in theory, a better thermal and chemical stability. Nonetheless, in the case of MOF-177,^18^ that possess the highest BET surface area, pore volume and the record of SO_2_ capture, the use of robust polynuclear cluster [Zn_4_(μ_4_-O)(O_2_CR)_6_] (see comparative Table 1), is not enough to achieve a reversible SO_2_ adsorption and the framework suffers a partial decomposition.

This result is most likely due to the propensity of the Zn–O bond to bind SO_2_ and form SO_3_^2−^. Similar behaviour was observed on a ZnO surface exposed to SO_2_ gas, that reacts with the oxygen atoms from the Zn–O bonds forming the SO_3_^2−^ and SO_4_^2−^ species.^22,23^ In fact, other Zn-based MOFs that have been studied for SO_2_ adsorption have shown similar stability problems. This is also the case of Zn-MOF-74 (ref. 24) with a rod-like SBU and coordinatively unsaturated zinc sites, where a Zn⋯O=S=O interaction leads to a chemisorption. Additionally, materials with paddlewheel SBUs, such as Zn^2+^-BDC (MOF-2) where the catalytic oxidation of SO_2_ under humid conditions was confirmed by the presence of sulphate species.^25^ Conversely, when the coordinatively unsaturated zinc site is blocked (*i.e.*, pillared MOFs), direct interaction of adsorbate with the metal centre is not observed, as for example in Zn(bdc)(ted)_0.5_ (ref. 26) and Zn-DMOF.^27^ These comparative results suggest that in the case of Zn-based materials, the robustness of the SBU plays a secondary role in chemical stability against SO_2_, and the fact that they are constructed from a divalent metal that forms labile bonds,^28^ makes them more susceptible to collapse.

Although MFM-170 contains the Cu^2+^ paddlewheel SBU, a cluster frequently instable under harsh conditions such as corrosive or acidic gases,^29,30^ it is stable even after 50 adsorption–desorption cycles of SO_2_.^19^ This extraordinary chemical stability is mainly due to two important factors: (i) the unusual paddlewheel SBU formed in the framework and (ii) additional intermolecular interactions formed between SO_2_ and the pore-walls of the framework. The di-cooper paddlewheel SBU shows the particularity of only one of the Cu^2+^ centres axially coordinated to a nitrogen atom from the pyridyl-based ligand, leaving the other Cu^2+^ metal centre coordinatively unsaturated and pointing toward the centre of the pore (Table 1). The effect of this structural arrangement is evident on the high SO_2_ uptake, where the Cu^2+^ sites are able to fix SO_2_ by forming a strong Cu–O bond without ligand displacement. Such behaviour was previously observed in a Cu^2+^-BDC MOF material.^25^ It is significant that this interaction does not represent the main SO_2_ binding site due to the steric hindrance generated by a neighbouring SO_2_ molecule. This latter molecule has several interactions: (i) with the C–H portion of the ligand, (ii) with the oxygen atoms in the paddlewheel SBU, and (iii) with other SO_2_ molecules. All these interactions promote an efficient packing within the pore surface, maximising the adsorbate–adsorbent bonds and presumably avoids the frame destruction. Both materials reviewed above, MOF-170 and MFM-177, are formed by first-row transition metals Zn^2+^ and Cu^2+^, respectively, and follow the stability order of the Irving–Williams series for divalent metals: Mn < Fe < Co < Ni < Cu > Zn.^31^ Thus, the high SO_2_ capture can be attributed to the large BET surface area and pore size rather than to the local pore environment, while structural stability is associated with the type of metal, and the interactions adsorbate–adsorbent.

Additionally, the use of higher-valent metal ions such as Al^3+^, Sc^3+^, Cr^3+^ or Zr^4+^ allows the formation of stronger M–O bonds with carboxylate ligands compared to divalent ions.^21^ Thus, the lability of metal–ligand bonding is higher in Cu^2+^ or Zn^2+^-based MOFs that Cr^3+^ MOFs. As a consequence, MOFs constructed from divalent metals such Cu^2+^ and Zn^2+^, typically show partial or total degradation under harsh conditions.^32^ Due to the inertness of the Cr–O bond, Cr^3+^-based MOFs highly resistant to the attack of acid and base guests were reported.^33,34^ As described above, the third material, MIL-101(Cr)-4F(1%), has the highest SO_2_ adsorption among SO_2_-stable MOFs.^20^ This framework poses a robust trinuclear metal cluster with coordinatively unsaturated chromium sites after the activation process (Table 1). When comparing MIL-101(Cr)-4F(1%) to MFM-170, both materials stable towards SO_2_, we can observe that the Cr^3+^ is harder than Cu^2+^ ions according to the Pearson acid–base concept^35^ and therefore the coordinated bond with carboxylic ligands is stronger for the trivalent cation than for the divalent cation. However, the Cu^2+^ paddlewheel increases its stability when the N-containing ligand coordinates to one of the Cu^2+^ ions.^36^

#### (ii) μ-OH groups

This particular hydroxo-functionalisation in MOF materials is well exemplified by the MFM-300(M) (MFM = Manchester Framework Material, M = Al^3+^, In^3+^, Ga^3+^ and Sc^3+^) family. These outstanding MOF materials are chemically stable towards SO_2_ and have been reported by Schröder and Yang.^37^ The general chemical formula of this group of MOFs is [M_2_(OH)_2_(L)] (H_4_L = biphenyl-3,3′,5,5′-tetracarboxylic acid = C_16_O_8_H_6_). MFM-300(M) shows a 3D open framework which contains [MO_4_(OH)_2_] octahedra connected *via* the *cis*-μ-OH groups into infinite chains, and further coordinated by the tetradentate ligand (L^4−^). This particular organisation produces highly porous materials (see Table 2), well defined by one-dimensional pore channels organized in a “wine rack” display.

**Table 2:** Comparison of MFM-300(M) family

Metal center (M)	BET Surface area [m^2^ g^−1^]	Pore volume [cm^3^ g^−1^]	SO_2_ adsorption [mmol g^−1^]	Packing density [g cm^−3^]	*Q*_st_ [kJ mol^−1^]	Stability
Al	1370	0.375	7.1	1.213	31.25^a^^37*e*^	Stable over 4 years of exposure
In	1071	0.419	8.28	1.266	34.5	Stable in dry and humid conditions
Ga	1045	0.62	—	—	—	—
Sc	1360	0.56	9.4	1.075	36.2	Stable in dry and humid conditions/over 10 cycles

a Adsorption near the zero-coverage calculated by GCMC.

**Fig. 2:**
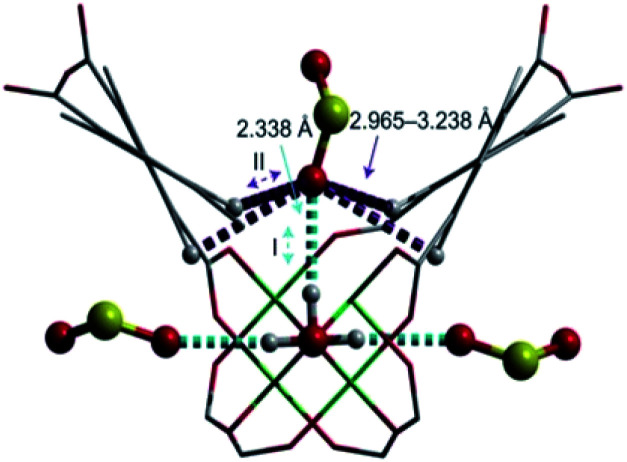
Preferred binding sites for SO_2_ molecules inside MFM-300(Al) determined by *in situ* PXRD. (I) Moderated hydrogen bond. (II) Weak hydrogen bond. Reprinted (adapted) with permission from ref. 37*a*. Copyright (2012) Springer Nature.

**Fig. 3:**
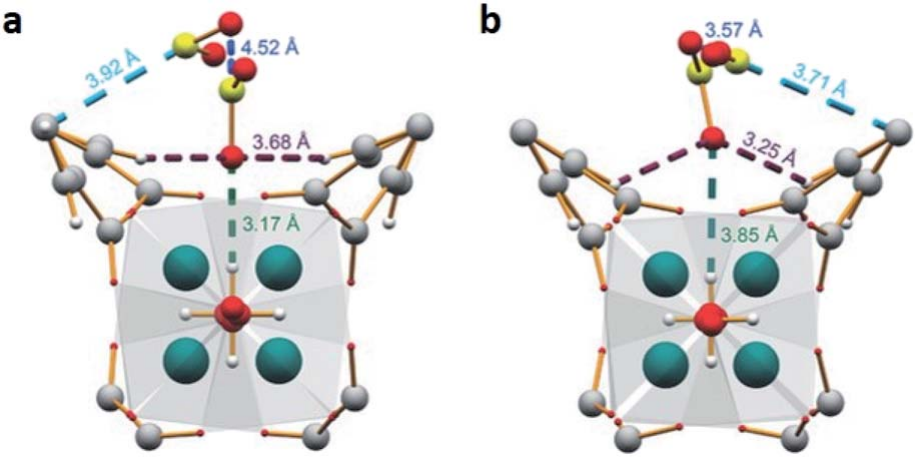
Binding sites for SO_2_ molecules within MFM-300(In) determined by single-crystal X-ray diffraction (a), and DFT calculations (b). Reprinted (adapted) with permission from ref. 37*b*. Copyright (2016) John Wiley & Sons.

The first MFM-300 material investigated for the capture of SO_2_ was MFM-300(Al).^37*a*^ Although the SO_2_ capture was not remarkably high (see Table 2), the identification of the preferential SO_2_ adsorption sites was achieved by sophisticated *in situ* INS and PXRD experiments, revealing that the μ-OH groups bind SO_2_ molecules through the formation of O=S=O(*δ*^−^)⋯H(*δ*^+^)–O hydrogen bonds, reinforced by weak supramolecular interactions with C–H atoms from the aromatic rings of the framework (Fig. 2).^37*a*^ In the case of MFM-300(In) (also known as InOF-1 (ref. 38)) the SO_2_ uptake was slightly higher than for MFM-300(Al) (see Table 2), and *in situ* INS and PXRD experiments corroborated the same preferential adsorption site for SO_2_ (see Fig. 3) with exceptional chemical stability for SO_2_ capture under both dry and humid conditions.^37*b*^ In fact, Eddaoudi and Salama fabricated an advanced chemical capacitive sensor, using MFM-300(In), for the detection of very low concentrations of SO_2_ (≈5 ppb) at room temperature.^39^

**Fig. 4:**
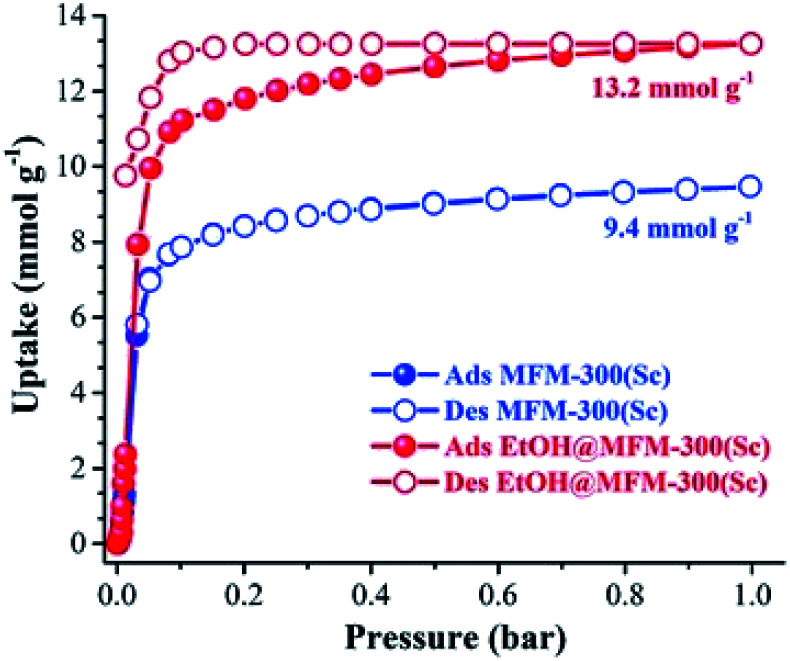
SO_2_ adsorption isotherm of scandium-based MFM-300 (blue isotherm) and after confinement of 2.8% EtOH (red isotherm). Reprinted with permission from ref. 37*c*. Published (2019) by The Royal Society of Chemistry.

MFM-300(Sc) showed the highest SO_2_ uptake of the MFM-300(M) materials (see Table 2), Grand Canonical Monte Carlo (GCMC) simulations demonstrated the same preferential adsorption sites (μ-OH) and by confining small amounts of EtOH (2.6 wt%) the SO_2_ capture increased by 40% (see Fig. 4).^37*c*^ The only MFM-300 remaining to be investigated in the capture of SO_2_ is MFM-300(Ga),^37*d*^ where we anticipate, based on the pore volume, performance similar to MFM-300(Sc).

Thus, the MFM-300(M) MOF materials have shown the relevance of the hydroxo-functionalisation to the capture and detection of SO_2_ with high chemical stability and excellent cyclability involving a remarkably facile regeneration. The key for all of such outstanding properties is the strength of the coordination bonds between the oxygen atoms form the carboxylic ligand (biphenyl-3,3′,5,5′-tetracarboxylic acid) and the M^3+^ metal centres. The ionic radii of Al^3+^, In^3+^ and Sc^3+^ cations, in octahedral coordination, are 0.675, 0.940 and 0.885 Å, respectively.^40*a*^

**Fig. 5:**
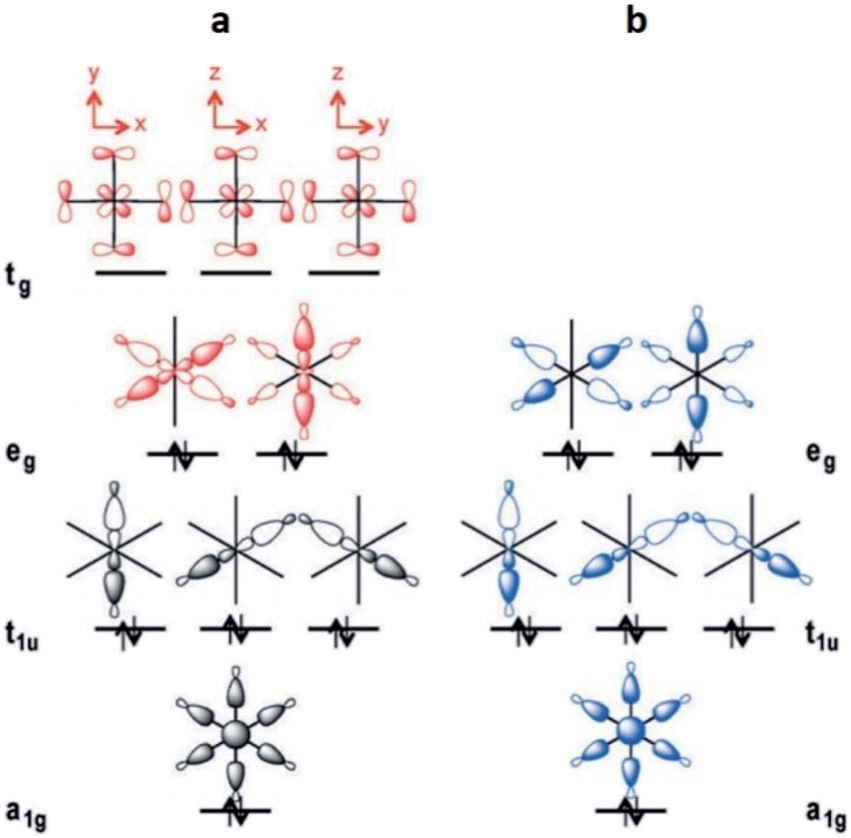
Schematic MO diagrams for an *O*_h_-symmetric 12e^−^ complex with d orbitals (a, red) and the MO diagram without d orbitals in 7c-12e^−^ pattern of MO (b, blue). Reprinted (adapted) with permission from ref. 40*c*. Copyright (2015) John Wiley & Sons.

This leads to a higher surface charge density for the Al^3+^ metal centre and, therefore, the M–O bond strength decreases in this order: Al^3+^ > Sc^3+^ > In^3+^. Thus, a higher ligand exchange can occur in the In-based complex.^40*b*^ However, MFM-300(In) does not suffer apparent displacement of carboxylate ligands after SO_2_ exposure. In the MFM-300(M) family all the metal cations are nonmagnetic because of all paired electrons,^41^ and the comparison between octahedral complexes formed by trivalent cations of group 13 such as Al^3+^, Ga^3+^ and In^3+^, with the transition-metal analogue Sc^3+^, has shown that in the group 13 metal complexes the d orbitals are not involved in the metal–linker bonding due to their high energy (3d^10^ and 4d^10^ configuration). In the case of Sc^3+^, a complex with 12e^−^, d orbitals form part of the M–O bonds (Fig. 5a). On the other hand, these types of complexes are considered as electron-rich hypervalent species with 7-center-12-electron bonding pattern (Fig. 5b).^40*c*^ This type of binding confers to the group 13 complexes thermal stability and in this particular case, the kinetic stability is comparable to that of the transition metal analogues. At this point, we can corroborate that frameworks constructed with trivalent cations are more stable than MOFs based on divalent cations (considering the stability of Irving–Williams series discussed above), as well that carboxylate ligands form with M^3+^ cations stronger bonds than divalent cations with N-based ligands.^32^

#### (iii) Defective sites

Crystal irregularities (composition inhomogeneities or defects) are fundamental characteristics of some solid-state materials and provide very particular physical and chemical properties.^42^ Such defects do not necessarily mean adverse effects. Due to their extraordinary modularity and tunability, MOFs allow the introduction of different kinds of defects while maintaining the overall structure integrity. Typically, “defective MOFs” have showed extraordinary superior catalysis performances,^43^ while Navarro and co-workers have taken a step forward and elegantly demonstrated the self-detoxification of defective UiO-66 examples to filter chemical-warfare agents.^44^ In the case of SO_2_, the same research group reported on the increase of the SO_2_ adsorption capacities, energies and SO_2_/CO_2_ selectivity for defective nickel pyrazolate MOF materials, prepared by introducing extra-framework Ba^2+^ ions into the porous structure and ligand functionalisation.^45^

**Fig. 6:**
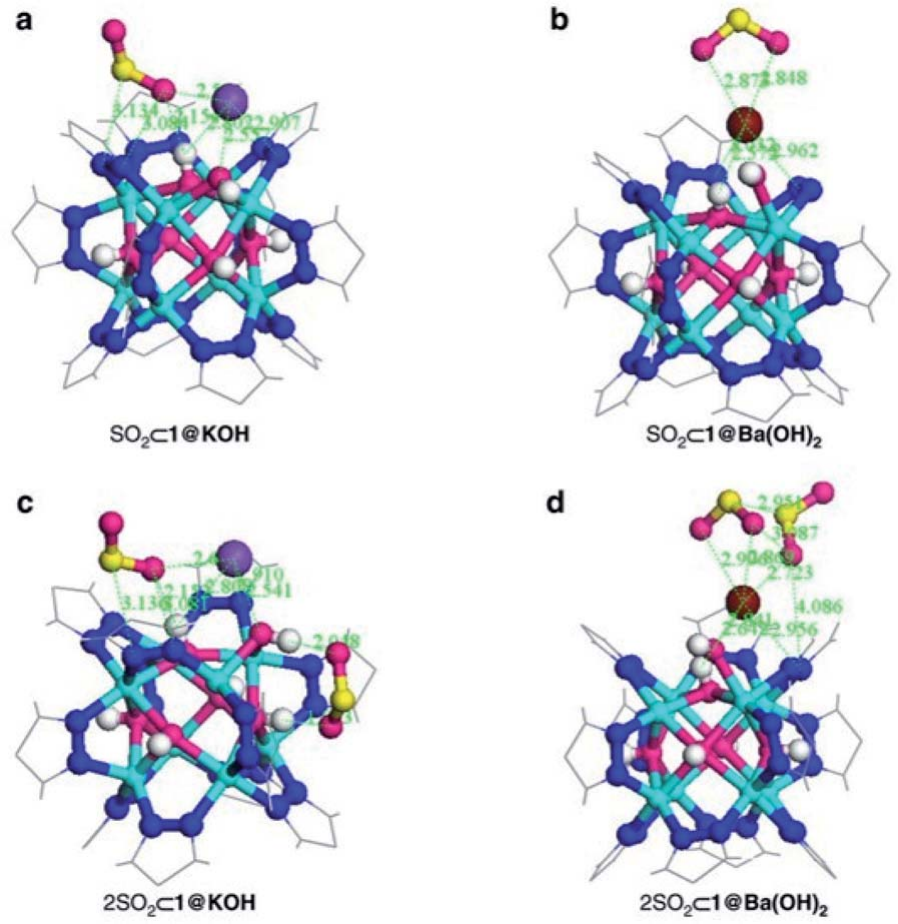
DFT structure minimization of molecular configuration of one (a and b) and two (c and d) adsorbed SO_2_ molecules on **1@KOH** (left) and **1@Ba(OH)2** (right) materials. For sake of clarity, only the region around the metal cluster is shown. Ni (cyan); K (purple); Ba (wine); C (grey); N (blue); O (magenta); H (white); S (yellow). Reprinted (adapted) with permission from ref. 45. Copyright (2017) Springer Nature.

Thus, the post-synthetic treatment of the [Ni_8_(OH)_4_(H_2_O)_2_(BDP_X)_6_] (H_2_BDP_X = 1,4-bis(pyrazol-4-yl) benzene-4-X with X = H (**1**), OH (**2**), NH_2_ (**3**)) systems with ethanolic solutions of potassium hydroxide leads to the formation of defective K[Ni_8_(OH)_3_(EtO)_3_(BDP_X)_5.5_] (**1@KOH**, **3@KOH**) and K_3_[Ni_8_(OH)_3_(EtO)(BDP_O)_5_] (**2@KOH**).^46^ Exposing the **1@KOH–3@KOH** materials to aqueous Ba(NO_3_)_2_ solutions incorporates barium cations into the solution, yielding the defective ion exchanged Ba_0.5_[Ni_8_(OH)_3_(EtO)_3_(BDP_X)_5.5_] (**1@Ba(OH)2**, X = H; **3@Ba(OH)2**, X = NH_2_), and Ba_1.5_[Ni_8_(OH)_3_(EtO)(BDP_O)_5_] (**2@Ba(OH)2**) systems.^45^ Interestingly, density functional theory (DFT) calculations located the extra-framework cations close to the crystal defect sites (Fig. 6a). In addition, DFT results demonstrated that the average 3D structure of the MOF framework is preserved.^45^ SO_2_ dynamic adsorption experiments confirmed the beneficial influence of the deliberate introduction of defects by a subsequent K^+^ to Ba^2+^ ion exchange process on the SO_2_ capture.^45^ The authors demonstrated that the pre-synthetic introduction of amino and hydroxyl functional groups on the organic linkers and the post-synthetic modifications, synergistically increase the SO_2_ capture capacity of these materials. In addition, chemisorption of SO_2_ was observed in all the systems: after all available sites for SO_2_ chemisorption were occupied, during the first exposure, reversible adsorption takes place in a steady way evidencing the stability of the materials upon continuous SO_2_ exposure. This high affinity of these MOF materials for SO_2_ was attributed to the increased basicity of the nickel hydroxide clusters after the introduction of defects. Lewis acid–base interactions with SO_2_ molecules can afford the formation of HSO_3_^−^ and SO_3_^2−^ species according to eqn (1):

1 [Ni_8_(OH)_6_(OH)_2_]^10+^ + SO_2_ → [Ni_8_(OH)_5_(HSO_3_)]^10+^ → [Ni_8_(OH)_4_(SO_3_)(H_2_O)]^10+^

Furthermore, the existence of extra-framework cations located close to defects sites promotes the formation of more stable MSO_3_ (M = Ba, 2 K) sulphite species according to eqn (2):

2 M[Ni_8_(OH)_4_(SO_3_)(H_2_O)(BDP_*X*_)_5.5_]^+^ → (MSO_3_)[Ni_8_(OH)_4_(H_2_O)(BDP_X)_5.5_]^+^

These chemisorption mechanisms were supported by the DFT simulations (Fig. 6), showing that **1@Ba(OH)2** establishes stronger interactions with SO_2_ molecules than **1@KOH** due to the specific interactions with extra-framework barium cations.

Thus, this sophisticated and comprehensive study, by Navarro *et al.*,^45^ carefully incorporated essential variables to improve the SO_2_ capture performance of these MOF materials. Such variables play together to increase the interaction with SO_2_ and can be summarised as (i) enhanced basicity of metal hydroxide clusters as a result of additional hydroxide anions replacing the missing linkers defects; (ii) affinity of extra-framework Ba^2+^ ions for SO_2_ sequestration; (iii) higher pore accessibility of the 3-D structure due to missing linker defects and (iv) the fine-tuning of the pore surface polarity by the benzene functional groups.

The post-synthetic inclusion of Ba^2+^ cations to improve the SO_2_ adsorption was later validated by Navarro and co-workers^47^ in the Ni-based MOF material Ni_2_^2+^{Ni_4_^2+^[Cu_4_^2+^–(Me_3_mpba)_2_]_3_}·54H_2_O (**1**). In this case, the exchange of the Ni^2+^ cations for Ba^2+^ was accomplished by soaking **1** in an aqueous solution of Ba(NO_3_)_2_ for two days to finally obtain a novel crystalline phase of heterotrimetallic oxamato-based MOF with the formula [Ba^2+^(H_2_O)_4_]_1.5_[Ba^2+^(H_2_O)_5_]_0.5_ {Ni_4_^2+^[Cu_2_^2+^–(Me_3_mpba)_2_]_3_}·57.5H_2_O (**2**). On this occasion, the presence of the hydrated Ba^2+^ counterions within the MOF's pores allowed the SO_2_ molecules to interact with the network reversibly *via* Cu^2+^⋯O_SO_2__ and O_H_2_O_⋯O_SO_2__ interactions. The amount of SO_2_ adsorbed in **2** increased from 2.0 to 2.5 mmol g^−1^ in comparison to **1**, respectively. This reversibility was conserved over 10 adsorption–desorption cycles. Theoretical calculations demonstrated that the principal binding site of SO_2_ molecules is with coordinated water molecules, and it showed moderate adsorption energy of −65.5 kJ mol^−1^, otherwise, the direct interaction of SO_2_ with Ba^2+^ cations would induce a higher adsorption energy (>100 kJ mol^−1^) due to the formation of an irreversible bond. Thus, this research group has elegantly proved that the formation of defective sites and the incorporation of large cations such as Ba^2+^ is a good and feasible post-synthetic method for modulating the adsorptive capacities of MOF materials.

#### (iv) Halogen functionalisation

Halogen functionalised materials are developing as promising technological platforms for innovative applications in different research fields such as catalysis, environmental remediation, sensing, and energy transformation.^48^ On the other hand, cationic or anionic frameworks have shown potential applications in the capture of gases as the ions present inside the pores of the MOFs can be a benefit for the interaction with guest molecules.^49^ The presence of halogen counterions in porous materials has demonstrated an enhancement of the SO_2_ capture performance due to the involvement of ionic bonding.^50^ The following section is devoted to MOFs with halogen ions in their structure that participate in the adsorption of gaseous SO_2_.

**Fig. 7:**
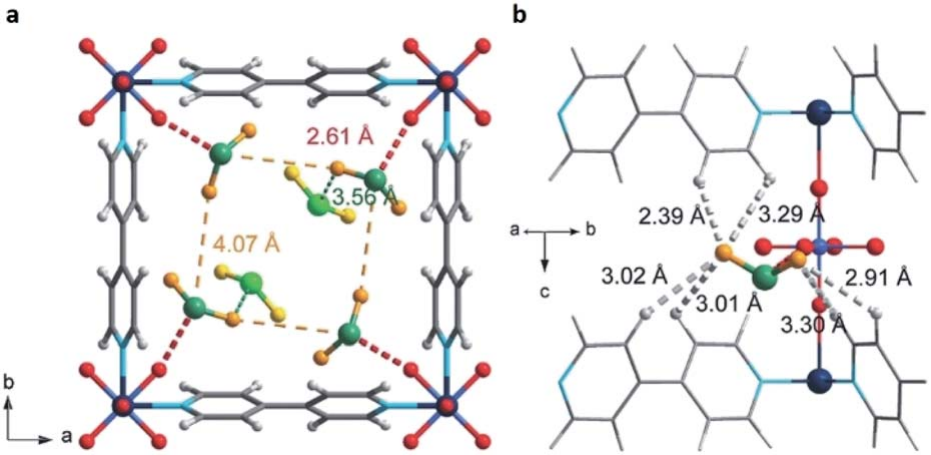
Binding sites for SO_2_ molecules adsorbed within SIFSIX-1-Cu determined by DFT-D calculations. Interaction S^*δ*+^⋯F^*δ*−^ (a), and dipole–dipole interactions with the ligand O^*δ*−^⋯H^*δ*+^ (b). Cu (dark teal); F (red); Si (light blue); C (grey); H (light grey); N (sky blue); O (orange); S (sea green). Reprinted (adapted) with permission from ref. 51. Copyright (2017) John Wiley & Sons.

Yang and Xing incorporated inorganic hexafluorosilicate (SiF_6_^2−^, SIFSIX) anions (as pillars) into a series of MOF materials (SIFSIX-1-Cu, SIFSIX-2-Cu (2 = 4,4′-dipyridylacetylene), SIFSIX-2-Cu-i, SIFSIX-3-Zn (3 = pyrazine), and SIFSIX-3-Ni) and investigated the SO_2_ adsorption properties of these materials.^51^ The remarkable highly efficient removal of SO_2_ from other gases, particularly at a very low SO_2_ concentrations, and excellent SO_2_/CO_2_ and SO_2_/N_2_ selectivities were attributed to the strong electrostatic interactions between the SO_2_ molecules and the SiF_6_^2−^ anions (S^*δ*+^⋯F^*δ*−^), assisted by dipole–dipole interactions with the ligand (O^*δ*−^⋯H^*δ*+^).^51^ The authors identified the interactions between the SO_2_ molecule and SIFSIX materials by modelling studies using first-principles DFT-D (dispersion-corrected density functional theory) calculations (see Fig. 7).^51^ These computational calculations were experimentally corroborated by Rietveld refinement of the powder X-ray diffraction patterns of SO_2_-loaded samples to locate the adsorbed positions of the SO_2_ molecules in the crystal structure of SIFSIX materials.^51^ Thus, this strategy highlighted the relevance on creating the multiple binding sites (anionic and aromatic linkers) which can originate the specific recognition of SO_2_ to optimise its capture.

**Fig. 8:**
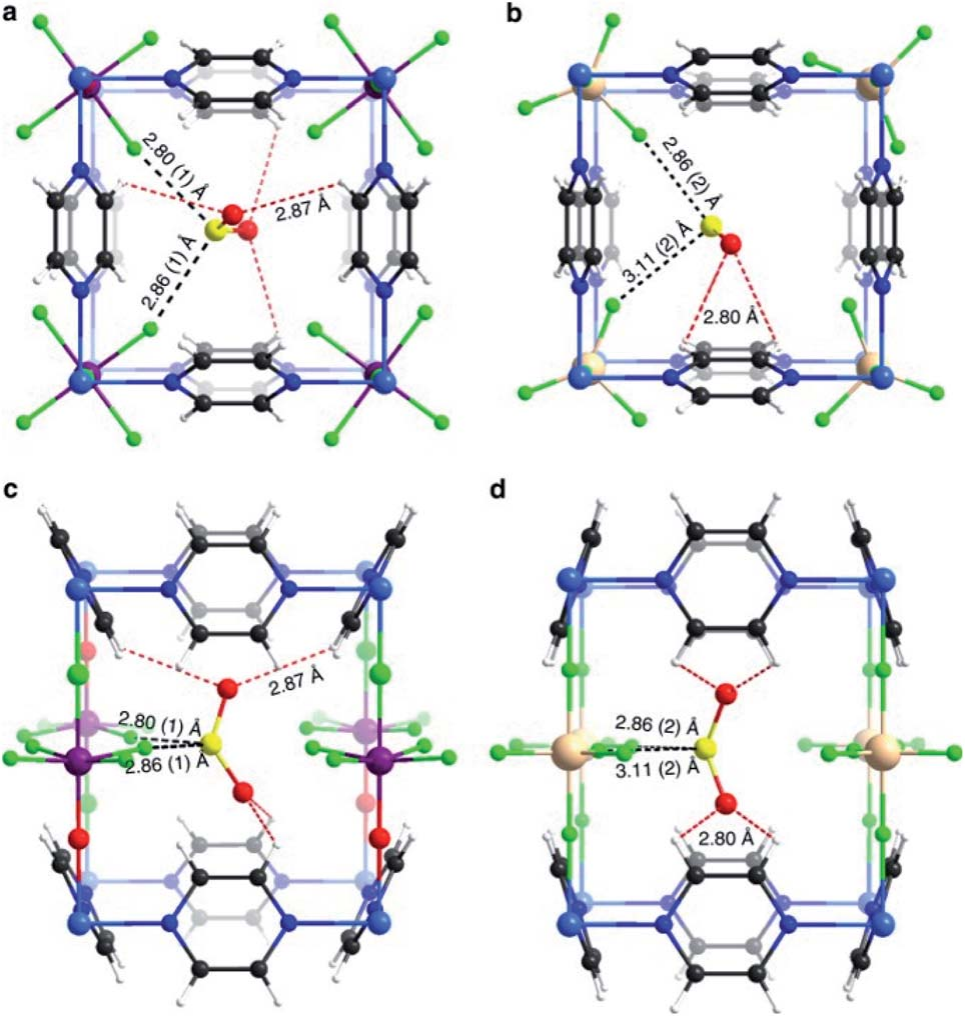
SO_2_ interactions within KAUST-7 (a and c), and KAUST-8 (b and d). Ni (blue light); Nb (purple); Al (beige); F (green); C (black); N (blue); O (red); H (white); S (yellow). Reprinted (adapted) with permission from ref. 52. Copyright (2019) Springer Nature.

Similarly, Salama and Eddaoudi demonstrated how fluorinated MOFs (KAUST-7 and KAUST-8) can be formidable candidates for sensing SO_2_ from flue gas and air (250 ppm to 7% of SO_2_).^52^ SCXRD data collected on the SO_2_-loaded KAUST-7 and KAUST-8 identified the preferential adsorption sites for SO_2_ revealing, comparable to SIFSIX materials, strong electrostatic interactions between the SO_2_ molecules and the fluorinated pillars (S^*δ*+^⋯F^*δ*−^), supported by dipole–dipole interactions with the pyrazine ligand (O^*δ*−^⋯H^*δ*+^), see Fig. 8.^52^ These interactions were also corroborated by DFT calculations.

Another remarkable halogen functionalisation was presented by Janiak and co-workers,^53^ where MOF-801 was modified by reacting zirconium halides (ZrX_4_; X = Cl, Br, I) in water with acetylenedicarboxylic acid. The HX addition and material construction occurred in a one-pot reaction yielding three microporous HHU-2-X MOFs (X = Cl, Br, I). The material HHU-2-Cl showed in increased SO_2_ uptake (by 21%) in comparison to the nonhalogenated MOF-801.^53^ Therefore, as previously demonstrated by the SIFSIX materials, KAUST-7, and KAUST-8, the electrostatic interactions between the SO_2_ molecules and the Cl (S^*δ*+^⋯Cl^*δ*−^), might be responsible for such SO_2_ capture enhancement.

## 3. Hydrogen sulphide

H_2_S is released to the environment *via* natural events such as volcanic eruptions, gas streams, hot springs, decomposition of organic matter and bacterial reduction.^54^ Additionally, H_2_S is emitted by some chemical industries, *e.g.*, oil desulphurisation processes at oil refineries, burning fossil fuels and mass transportation.^55^ H_2_S is a main air pollutant which negative impacts the environmental as it is one of the main sources of acid rain,^55^ and it is highly toxic to humans (concentrations above 700 ppm in the air can cause death).^56^

H_2_S is a colourless, flammable gas with a characteristic rotten egg odour. Typical strategies to capture H_2_S comprise alkanolamines and ionic liquids, adsorption in solid materials (*i.e.*, zeolites, activated carbons and metal oxides), membrane separation and cryogenic distillation.^57^ However, these techniques have major drawbacks, such as low H_2_S capture, corrosion of pipelines and large cost of expenditure and recovery.^58^ Thus, the development of new technologies for a dry adsorption process (avoiding solvents/water consumption is essential to reduce waste generation), for removal and capture of H_2_S signifies a technological challenge which could allow not only the capture of H_2_S from the main source, but also its use as feedstock, similarly to the CO_2_ technologies (CCS).^59^ MOFs have been visualised for the capture of H_2_S; however, most of them have shown poor chemical stability in its presence.^60^ Nonetheless, chemically stable MOF materials have demonstrated promising results in the reversible capture of H_2_S.^61^ This section highlights outstanding MOF materials that have shown high H_2_S capture performances and *in situ* H_2_S transformations, as well as biomedical applications.

### 3.1 MOFs for H_2_S capture

Distinctive investigations reported by De Weireld,^62^ Zou^63^ and Eddaoudi^64^ reveal that the majority of MOF materials experience structural decomposition upon adsorption of H_2_S or the desorption of it resulted complicated, due to relatively strong host–guest binding in the pores (strong physisorption or chemisorption). In such cases, the reactivation of the materials involves a large energy penalty or is not feasible. Thus, the identification of new MOFs capable of capturing H_2_S under industrially practical pressure-swing desorption conditions,^65^ represents a very important challenge to solve. This perspective focused on (i) high and reversible H_2_S capture in MOFs and (ii) chemical transformation of H_2_S within the pores of MOFs: formation of polysulphides.

#### (i) High and reversible H_2_S capture in MOFs

The capture of H_2_S by MOFs has confirmed some crucial difficulties such as the formation of strong bonds, typically irreversible, (*e.g.*, a metal–sulphur bond), which can compromise the chemical stability and cyclability of MOFs. Therefore, it is required to modulate host–guest interactions between the MOFs and H_2_S to avoid structure collapse and afford a feasible cyclability. In order to achieve these goals, such interactions should arise through noncovalent bonding between the functionalisation of MOFs and H_2_S guest molecules. For example, hydrogen bonding has been postulated, by density functional theory (DFT) methods and grand canonical Monte Carlo (GCMC) simulations,^66^ as a promising moderate interaction between MOFs and H_2_S molecules. Experimentally, a remarkable work by Hamon and co-workers showed the high chemical stability and cyclability of MIL-47 (M = V^4+^) and MIL-53(M) (M = Al^3+^, Cr^3+^) when exposed to H_2_S.^67^ These MOF materials are constructed with μ-OH functional groups which presumably established hydrogen bonds with H_2_S.

**Fig. 9:**
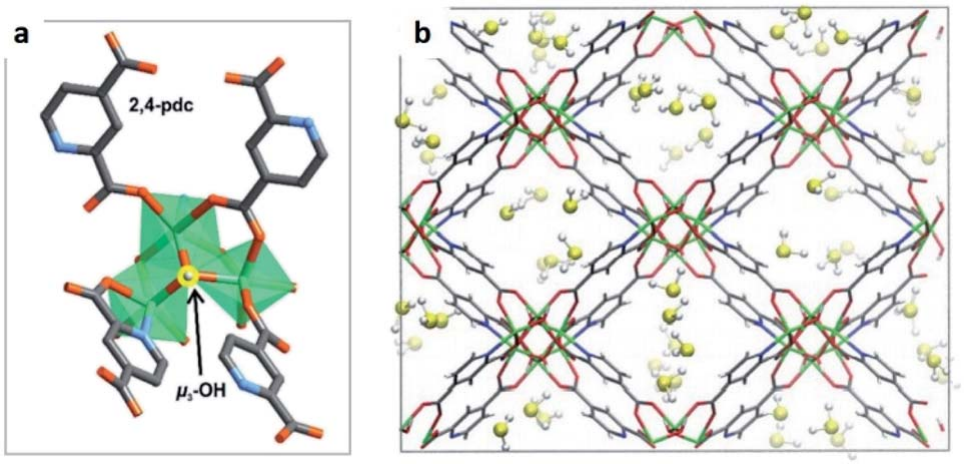
H_2_S adsorption preferred site of magnesium-based CUK-1 material. And after confinement of 2.8% EtOH (red isotherm). Reprinted (adapted) with permission from ref. 68. Published (2018) by The Royal Society of Chemistry.

Humphrey and Maurin showed the H_2_S capture on Mg-CUK-1.^68^ This MOF is assembled from a 2,4-pyridinecarboxylate ligand and Mg^2+^ octahedral centres, connected into infinite chains of [Mg_3_(μ_3_-OH)]^5+^ clusters by μ_3_-OH groups. Although the total H_2_S capture of Mg-CUK-1 was relatively low (3.1 mmol g^−1^), this material demonstrated to be chemically stable to H_2_S and a remarkably easy regeneration after the H_2_S capture using temperature swing re-activation (TSR). Most importantly, GCMC simulations showed how H_2_S molecules interact with the hydroxo functional groups (by moderate hydrogen bonds μ_3_-O–H⋯SH_2_), see Fig. 9.^68^ Such moderate H_2_S/Mg-CUK-1 interaction was found to be consistent with easy regeneration of the material after H_2_S exposure.

This work demonstrated experimentally and in good correlation with advanced computational calculations, the chemical structure integrity of a hydroxo-functionalised MOF material to H_2_S, and the critical role of a moderate interaction (hydrogen bonding) to facilitate its cyclability. The study of more μ-OH functionalised MOFs for the efficient and cyclable capture of H_2_S initiated.

**Fig. 10:**
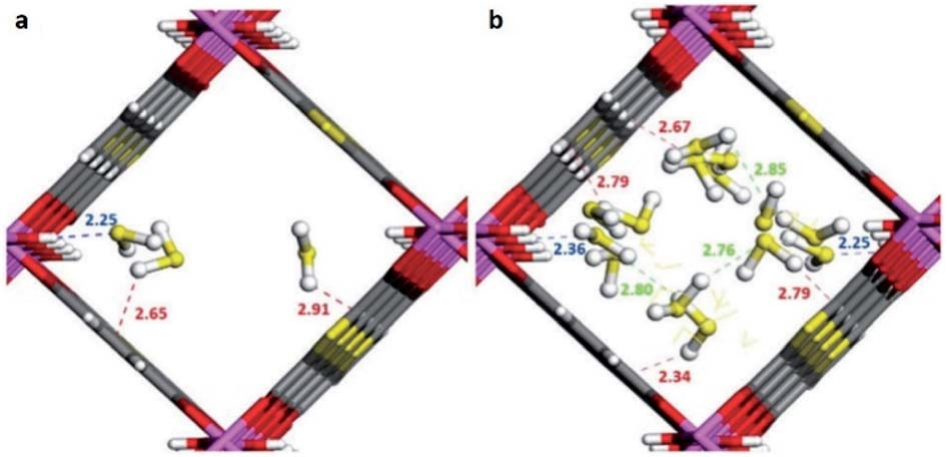
Illustrative arrangements of H_2_S in the pores of MIL-53-TDC generated from the GCMC simulations at (a) 0.5 mmol g^−1^ and (b) 18.5 mmol g^−1^. The distances are reported in Å. (Al, pink; O, red; S, yellow; C, grey; H, white). Reprinted (adapted) with permission from ref. 69. Published (2019) by The Royal Society of Chemistry.

Maurin and Gutiérrez-Alejandre investigated another μ-OH functionalised MOF (MIL-53(Al)-TDC) for the capture of H_2_S.^69^ This material, an Al^3+^-based constructed with a carboxylate ligand (TDC = 2,5-thiophenedicarboxylate) which contains [AlO_4_-*trans*-(μ-OH)_2_] octahedra where the Al^3+^ centre coordinates to two μ-OH groups and six oxygen atoms from the TDC ligands, has established the highest H_2_S adsorption (18.1 mmol g^−1^ at room temperature) reported for a microporous material.^69^ Structural stability of MIL-53(Al)-TDC, after the H_2_S capture experiment was corroborated by PXRD and SEM analyses. TGA experiments demonstrated the complete desorption of H_2_S molecules at 65 °C. H_2_S sorption–desorption cycles (five cycles with a value of 18.5 ± 0.7 mmol g^−1^) demonstrated high H_2_S regeneration capacity. This cyclability confirmed the full regeneration of the material by only increasing the temperature to 65 °C, showing the low energy requirement to fully desorb H_2_S. This suitable cyclability indicated weak interactions between H_2_S molecules and the pores of the material. *In situ* DRIFT experiments investigated the interactions between H_2_S and MIL-53(Al)-TDC, showing (i) the formation of hydrogen bonds between H_2_S molecules themselves confined in the pores of MIL-53(Al)-TDC; (ii) relatively weak interaction between H_2_S and the μ-OH groups and (iii) H_2_S molecules interact with the other functionality of the MOF: the thiophene ring from the TDC ligand.^69^ To corroborate these interpretations from the DRIFT experiments, Monte Carlo simulations were carried out for different loadings corresponding to the experimental discoveries. These calculations corroborated that at low loading, H_2_S interacts *via* its S-atom with the H-atom of the μ-OH group (see Fig. 10), representing the preferential adsorption site. In addition, it was shown that H_2_S also interacts with the thiophene ligand, and, upon increasing the H_2_S loading, hydrogen bonds between the H_2_S molecules were also identified (Fig. 10).^69^

These theoretical findings revealed the fundamental role of the thiophene ligand. Experimentally speaking, this M–OH hydroxo group containing functional material shows an extraordinarily facile re-activation leading to an easy H_2_S cyclability, which should not be anticipated due to the dominant interaction between the μ-OH group and H_2_S reenforced further by the interaction of the H_2_S molecule with the thiophene unit as demonstrated by *in situ* DRIFT measurements. However, computational calculations postulate that the interaction of H_2_S with the thiophene ring weakens the interaction with the preferential adsorption site (μ-OH group), favouring the easy displacement of the H_2_S molecule when the material is re-activated.

As previously discussed, (*vide supra*) for the MFM-300(M) family, the extraordinary chemical stability arises from the strength of the coordination bonds between the oxygen atoms form the carboxylic ligand and the M^3+^ metal centres. Thus, MIL-53(Al)-TDC is very similar to MFM-300(Al) since it is also constructed with Al^3+^. Therefore, the chemical stability of MIL-53(Al)-TDC towards H_2_S is envisaged based on the same arguments used for MFM-300(Al).

#### (ii) Chemical transformation of H_2_S within the pores of MOFs: formation of polysulphides

As described in the previous section, the study of more hydroxo-functionalised MOF materials attracted high interest since the promising results on the effective and efficient cyclable capture of H_2_S. Thus, a couple of materials from the MFM-300(M) family were investigated by Maurin and Gutiérrez-Alejandre,^70^ for the capture of H_2_S: MFM-300(Sc) and MFM-300(In).

First, MFM-300(Sc) exhibited a H_2_S uptake of 16.5 mmol g^−1^ (at 25 °C), which is comparable to the H_2_S uptake of MIL-53(Al)-TDC (18.1 mmol g^−1^).^69^ Upon an inspection of the structural integrity of the material after the H_2_S experiment, PXRD experiments revealed the retention of the crystalline structure. Interestingly, when investigating the porosity of MFM-300(Sc) after the H_2_S uptake experiment, the pore volume was reduced by 34% from 0.56 to 0.37 cm^3^ g^−1^ indicating that some remaining species are still present in the pores of the material. After confirming the retention of the crystallinity and a reduction of the intrinsic porosity of MFM-300(Sc) after the first H_2_S cycle, additional H_2_S cycling experiments were performed. Over the second cycle, the H_2_S adsorption capacity decreased by 39% to 10.08 mmol g^−1^, which is in a good agreement with the reduction of the pore volume by about 34%. The average H_2_S capture in cycles 2 to 5 was 10.22 mmol g^−1^. After the fifth cycle, PXRD experiments corroborated the retention of the crystalline structure of MFM-300(Sc), while a N_2_ adsorption experiment showed that the pore retained its reduced volume of 0.37 cm^3^ g^−1^. In an attempt to remove the remaining sulphur species formed inside the pores of MFM-300(Sc), the sample after the first H_2_S cycle was activated at 250 °C. After this thermal treatment, the pore volume did not change (0.37 cm^3^ g^−1^) confirming that the sulphur species were irreversibly adsorbed within the MFM-300(Sc) framework.^70^

**Fig. 11:**
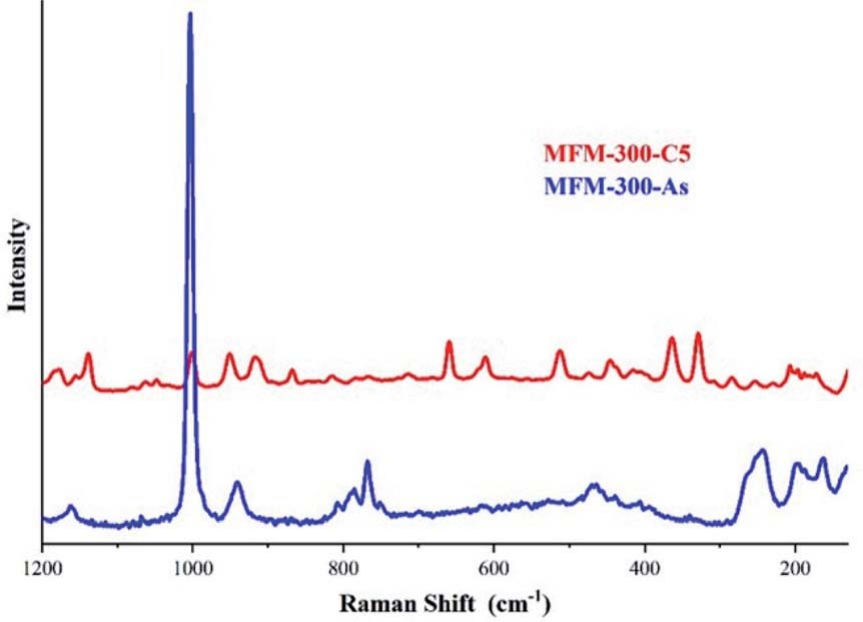
Raman spectra of MFM-300(Sc) after 5 cycles of H_2_S (red) and as synthesised (blue). The peaks observed in the 350–520 cm^−1^ region are associated to the S–S stretching vibrations modes of several polysulphides with various chain lengths. Reprinted with permission from ref. 70. Copyright (2020) American Chemical Society.

The identification of these sulphur species was first approached by TGA and diffuse-reflectance infrared Fourier-transform spectroscopy (DRIFT) experiments which corroborated strong interactions between the guest species and the pore-walls of MFM-300(Sc). Raman spectroscopy, complemented with elemental analysis (EDX) and conventional elemental analysis, postulated the first indication of the possible nature of the remaining (irreversibly adsorbed) sulphur species within the pores, *i.e.*, polysulphides (see Fig. 11).^70^ Even though the redox properties of polysulphides are highly complex, electrochemical experiments were the key to fully identify them. An electrochemical cell (MFM-300(Sc))-CSP/PVDF/1 M LiTFSI in triglyme/Li0 (PVDF = poly(vinylidene difluoride), LiTFSI = LiN[SO_2_CF_3_]_2_, triglyme = MeO[CH_2_CH_2_O]_3_Me) was examined (for 20 h) through open-circuit potential (OCP) measurements and analysed by cyclic voltammetry. Then, this electrochemical cell showed an initial potential of 2.29 V (*vs.* Li^0^/Li^+^) for MFM-300(Sc) and after reaching equilibrium, this potential was equal to 2.15 V (*vs.* Li^0^/Li^+^).

Later, a H_2_S exposed sample of MFM-300(Sc) (H_2_S@MFM-300(Sc)) was used to construct a different electrochemical cell showing a different behaviour on the variation of the potentials (the potential increased from 1.90 to 2.33 V (*vs.* Li^0^/Li^+^)). According to Mikhaylik and Akridge,^71^ electrochemical potentials lower than 2.10 V correspond to low-order polysulphides (S_*n*_^2−^), (*n* = 2). Thus, the initial potential for H_2_S@MFM-300(Sc) of 1.90 V suggested the formation of such low-order S_2_^2−^ polysulphides. The formation of these low-order species arises from the strong mutual H_2_S⋯H_2_S hydrogen bond interactions with a characteristic H(H_2_S)⋯S(H_2_S) distance of 2.91 Å, as demonstrated in the corresponding RDF plot from the Monte Carlo simulations.

In the case of MFM-300(In) by constructing the equivalent electrochemical cell (MFM-300(In))-CSP/PVDF/1 M LiTFSI in triglyme/Li^0^ and same operation conditions (OCP measurements and analysed by cyclic voltammetry for 20 h), the electrochemical cell exhibited an initial potential of 2.65 V (*vs.* Li^0^/Li^+^) for MFM-300(In) and after reaching equilibrium, this potential was equal to 2.15 V (*vs.* Li^0^/Li^+^). The difference of 0.36 V, from the initial potentials for MFM-300(Sc) and MFM-300(In), was attributed to the difference in the electronegativity of the metal centres (Sc^3+^ and In^3+^) in both materials. After the adsorption of H_2_S, H_2_S@MFM-300(In), MFM-300(In) showed a different trend than MFM-300(Sc): the potential decreased from 2.50 to 2.15 V (*vs.* Li^0^/Li^+^). Such a trend difference for both stabilisation potentials and the potential values indicated that the chemical composition of the sulphur species in both materials (MFM-300(Sc) and MFM-300(In)) were different. Again, Mikhaylik and Akridge indicate,^71^ that electrochemical potentials higher than 2.10 V correspond to high-order polysulphides (S_*n*_^2−^), (*n* = 6). Such large polysulphides block completely the pores of MFM-300(In), as corroborated by the loss of the pore volume in H_2_S@MFM-300(In) (0.02 cm^3^ g^−1^) and Raman spectroscopy. Interestingly, although these two materials are isostructural, a small difference in the pore size (8.1 and 7.5 Å for MFM-300(Sc) and MFM-300(In), respectively) contributes along with the different electrochemical potential of both materials (different electron densities) to the formation of distinct polysulphide species.

Although there is a hypothesis that justifies the polysulphide formation in both materials (based on the “disproportionation” type reaction,^72^ were the protons of H_2_S play the role of an oxidant and the sulphide plays the role of a reducing agent), there is a great opportunity to deeply investigate this phenomenon. For example, can the polysulphide formation be a consequence of reversible metal–ligand bonding upon the adsorption of H_2_S? Recently Brozek and co-workers^73^ elegantly demonstrated metal–ligand dynamics for carboxylate benchmark MOFs, *via* variable-temperature diffuse reflectance infrared Fourier transform spectroscopy (VT-DRIFTS) coupled with *ab initio* plane wave density functional theory. Thus, new exciting horizons can be discovered by taking a different approach when H_2_S is adsorbed in MFM-300(M), which could explain such fascinating polysulphide formation.

The last MOF material, to date, that demonstrated the chemical transformation of H_2_S to polysulphides is SU-101.^74^

**Fig. 12:**
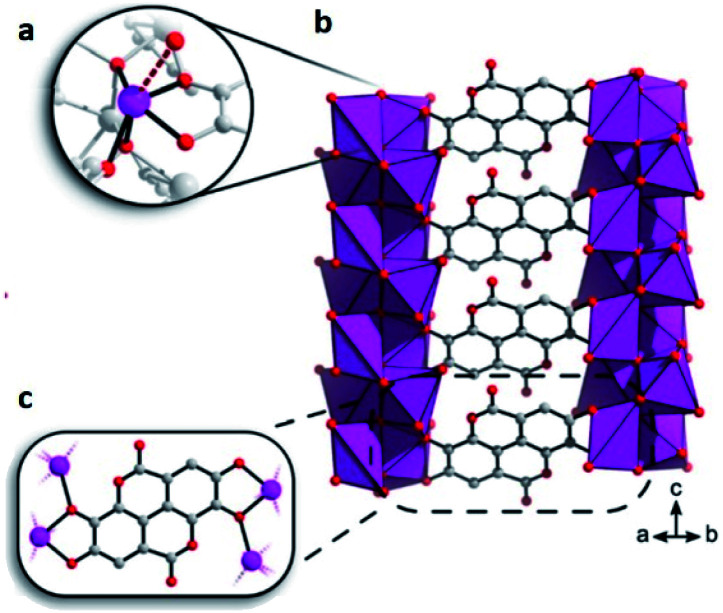
(a) The coordination environment around Bi^3+^. The bond to a coordinated water molecule is represented as a dashed line. (b and c) Chelation of ellagate ligand toward the bismuth oxo rods. Reprinted (adapted) with permission from ref. 74. Copyright (2020) American Chemical Society.

This bioinspired material was synthesised (by Inge *et al.*^74^), using ellagic acid, a common natural antioxidant, and bismuth (Bi_2_O(H_2_O)_2_(C_14_H_2_O_8_)·*n*H_2_O), see Fig. 12. Then, the resultant breakthrough H_2_S experiment led to a gas uptake of 15.95 mmol g^−1^, representing one of the highest H_2_S uptakes reported and even more interesting since the BET surface area of SU-101 (412 m^2^ g^−1^) is considerably lower than top H_2_S capture materials (*e.g.*, MIL-53(Al)-TDC; BET = 1150 m^2^ g^−1^). On a second H_2_S adsorption cycle, the initially observed capacity and surface area were reduced to only 0.2 mmol g^−1^ and 15 m^2^ g^−1^, respectively, even though the crystallinity was retained as confirmed by PXRD. Raman spectra of SU-101 (before and after H_2_S adsorption) confirmed the presence of low-order polysulphides (*n* = 2), S_4_^2−^. Thus, the electrochemical potential of SU-101 should be similar to the one for MFM-300(Sc) (2.29 V),^70^ considering that S_4_^2−^ species were only found for MFM-300(Sc). Future investigations are anticipated to continue in order to verify the electrochemical potential of SU-101 and any possible reversible metal–ligand bonding upon the adsorption of H_2_S.

A variety of MOFs, as well as classic materials such as zeolites, activated carbon and metal oxides, have been studied for the adsorption of these toxic gases, where some of these are summarized in Table 3. Noteworthy, the comparison of materials for H_2_S capture is sometimes challenging due to the difference in the experimental conditions (*e.g.*, flue gas concentration).

**Table 3:** Summary of the adsorption capacity of H_2_S for selected metal–organic frameworks and benchmark materials

MOF	BET Surface area [m^2^ g^−1^]	H_2_S adsorption [mmol g^−1^]	Ref.
MIL-47(V)	930	14.6	67
MIL-53(Cr)	1946	12.0	67
MIL-53(Al)-BDC	1103	11.7	67
Ni-CPO-27	1547	12.0	85
Mg-CUK-1	604	3.1^a^	68
MIL-53(Al)-TDC	1150	18.5	69
MIL-100(Fe)	2000	16.7	67
MIL-101(Cr)	2916	30.1	34
MIL-101(Cr)-4F(2%)	2176	36.9	34
MFM-300(Sc)	1350	16.5	70
Cu-BTC	1590	1.1	63
SU-101	412	15.95	74
PAF-302	5600	51.94	75
COF-102	3621	35.57^b^	75
HKUST-1^c^	—	2.7^d^	60
10Mn–45Zn–45Ti–O^e^	—	0.0005	76
4A molecular sieve zeolite	49.5	0.0002^f^	77
13X zeolite	440	0.007^g^	78
SBA-15	950	1.98^h^	79
PEI(50)/SBA-15(MBS-2)	80	0.0002^h^	80
Wood-based AC	1400	0.008	81

a 15% vol H_2_S.

b GCMC simulations.

c Graphite-oxide composite.

d 1000 ppm H_2_S in moist air.

e H_2_S/H_2_/CO_2_/H_2_O (600 ppm : 25% : 7.5% : 1%).

f 10% vol H_2_S.

g 80 ppmv H_2_S.

h 4000 ppmv of H_2_S and 20 vol% of H_2_ in N_2_.

### 3.2 Bio-compatible MOFs for H_2_S detection and controlled delivery

Although H_2_S is catalogued as highly toxic, paradoxically, it can also be crucial as an endogenous biological mediator.^82^ Endogenous pathways for H_2_S have been found to have wide extending activities *in vivo*, such as in the cardiovascular system.^83^ For example, H_2_S executes a crucial activity in the regulation of blood pressure, neurotransmission, anti-inflammatory mechanism, anti-oxidation, angiogenesis and apoptosis.^84^ H_2_S can be biosynthesised by enzymatic reactions *via* a sequence of endogenous processes,^85^ and in the biological medium, the H_2_S concentration can fluctuate from nano-to micromolar levels.^86^ However, at higher concentrations of H_2_S in the bloodstream can cause severe physiological disorders such as diabetes, Alzheimer's disease, cirrhosis, different types of cancer and Down's syndrome.^87^

Therefore, in order to recognise the specific role of H_2_S in these processes, the detection (spatial and temporal information) of H_2_S levels in living cells and organisms is crucial. However, due to the high reactivity, volatility and diffusible properties of H_2_S, traditional detection technologies (chromatography, high performance liquid chromatography, electrochemical analysis and colorimetric)^88^ are unsuitable for the identification of H_2_S in living cells because they result in destruction of the cells and tissues and difficulties associated to real-time detection.^89^ Fluorometric detection techniques have emerged as highly significant alternatives since their non-destructive characteristics, fast response, high selectivity and sensitivity, fast response, high spatial sampling capability, real-time monitoring and easy sample preparation.^90^ Turn-on type fluorescence probes for H_2_S detection have been recently investigated and these are commonly based on the reactivity characteristics of H_2_S (*e.g.*, copper sulphide precipitation, dual nucleophilic addition, H_2_S-mediated hydroxyl amide and nitro/azide reduction).^91^ Such characteristic reactivity of H_2_S can efficiently distinguish it from other biological species; however, many of these detectors do not fulfil basic requirements such as rapid detection and selectivity. Thus, the investigation of new H_2_S probes is still very attractive due of the complexity of different molecular events involved in signalling transduction, and other complications related to the probes. Therefore, bio-compatible MOFs have been postulated as promising probes for the detection of H_2_S.

The first concept of a MOF material in *in vitro* H_2_S delivery was demonstrated by Morris and co-workers who used CPO-27 for this purpose.^92^ H_2_S can be released by exposing the material to H_2_O since CPO-27 is an open metal site system thus, the water molecule replaces the hydrogen sulphide molecule at the metal site. The authors analysed the H_2_S delivery in two isostructural materials (Zn-CPO-27 and Ni-CPO-27) finding that the Ni^2+^ material exhibited higher delivery capacity than the Zn^2+^ material: after 30 min 1.8 mmol g^−1^ of H_2_S was released from Ni-CPO and only 0.5 mmol g^−1^ from the Zn-CPO. After 1 h, the H_2_S release for both materials was completed. The amount of H_2_S released by each material corresponds to approximately 30% of the chemisorbed gas. Later, the release of H_2_S (stored in Zn-CPO-27 and Ni-CPO-27) was investigated under physiological conditions (endothelium-intact ring of pig coronary artery), finding a substantial vasodilatory action (relaxation) with only a short induction period of approximately 5 min.^92^ These results represented a very significant progress in the field of MOFs and biological applications, since such H_2_S release mechanisms offered new protocols to better understand the effects of H_2_S on this particular vascular system.

**Fig. 13:**
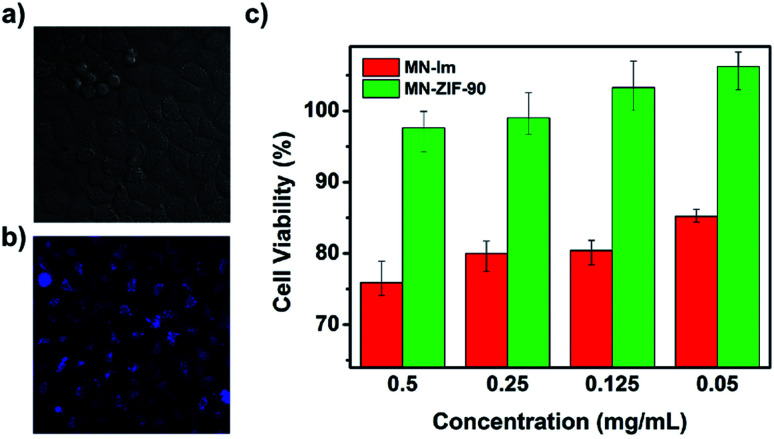
(a, b) Bright field image and fluorescence image after treating the HeLa cells with MN-ZIF-90 (0.05 mg mL^−1^) after 48 h of incubation. (c) *In vitro* HeLa cell viabilities after 48 h of incubation with MN-IM and MN-ZIF-90 at different concentrations. Reprinted with permission from ref. 93. Copyright (2014) Springer Nature.

Later, the first fluorescence probe example based on a MOF for the detection of H_2_S was presented by Wang and co-workers.^93^ Through a post-synthetic modification of ZIF-90 with malononitrile (N≡C–CH_2_–C≡N), they obtained MN-ZIF-90 which undergoes a specific reaction with H_2_S, achieving an enhancement of photoluminescence, constituting the base for the detection of H_2_S (see Fig. 13). Then, the malononitrile moieties conjugated to the host (ZIF-90) through double bonds aided as quenchers of the host fluorescence through intramolecular photoinduced electron transfer. The detection mechanism was based on the α,β-unsaturated bond of the malononitrile which is susceptible to thiol compounds, leading to the breaking of the double bond and thus, the fluorescence of MN-ZIF-90 was recovered once H_2_S was introduced into the system. In addition, the probe exhibited favourable biocompatibility.^93^

**Fig. 14:**
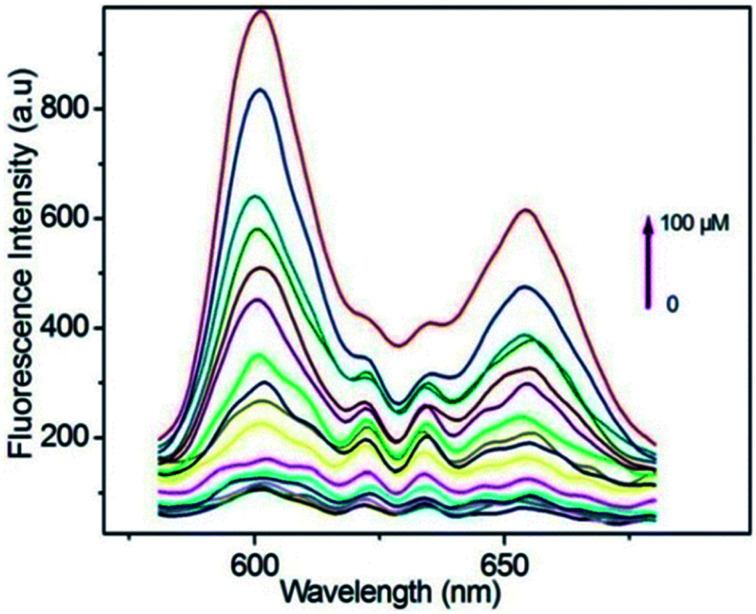
Fluorescence spectra of porphyrin ligand (10 μM) in BBS butter (pH = 7.40, 20 mM) after titration with HS^−^ from 0–100 μM, *λ*_ex_ = 419. Reprinted with permission from ref. 94. Copyright (2014) American Chemical Society.

Taking a different approach but keeping the fundamental idea of introducing a reactive site for H_2_S, Tang *et al.*,^94^ demonstrated the use of a porphyrin-based MOF for the fluorescent detection of H_2_S. By introducing reactive Cu^2+^ metal centres into a Al^3+^ MOF material {CuL[AlOH]_2_}_*n*_ (H_6_L = mesotetrakis(4-carboxylphenyl) porphyrin) the detection of H_2_S was selectively followed by fluorescence under physiological pH (see Fig. 14). Additionally, they successfully demonstrated the capability of the probe to detect exogenous H_2_S in living cells, while the probe showed low toxicity and high biocompatibility.^94^

**Fig. 15:**
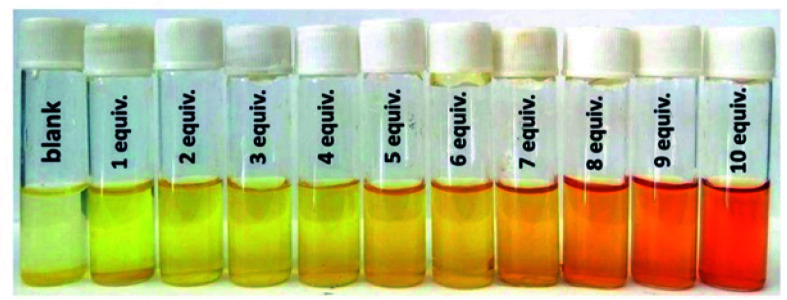
Colorimetric detection behaviour of the HEPES suspension of UiO-66-(NO_2_)_2_ with increasing concentration of Na_2_S in daylight. Reprinted with permission from ref. 95. Published (2018) by The Royal Society of Chemistry.

Later, Biswas and co-workers demonstrated, on a dinitro-functionalised UiO-66 (UiO-66-(NO_2_)_2_), fluorescence turn-on behaviour towards H_2_S in simulated biological medium (HEPES buffer, pH = 7.4).^95^ Remarkably, UiO-66-(NO_2_)_2_ exhibited highly sensitive fluorometric H_2_S sensing while also showing a visually detectable colorimetric change to H_2_S in daylight (see Fig. 15). In addition, the high selectivity of this functionalised MOF material to H_2_S was preserved even when several other biological species were present in the detecting medium. Finally, fluorescence microscopy studies on J774A.1 cells demonstrated the effectiveness of UiO-66-(NO_2_)_2_ for H_2_S imaging in living cells, detection of H_2_S in human blood plasma (HBP) and monitoring of the sulphide concentration in real water samples.^95^ These results emphasised the biocompatibility of UiO-66-(NO_2_)_2_. The reaction mechanism of UiO-66-(NO_2_)_2_ and H_2_S is caused by the reduction of the nitro groups to electron donating amine groups causing an increase in the fluorescence intensity that can be registered.

Eu^3+^/Ag^+^@UiO-66-(COOH)_2_ (EAUC) composites were reported by Li and Qian as biomarkers for the for potential diagnosis of asthma.^96^ The decrease of the H_2_S production in the lung has been identified as an early detection biomarker for asthma. Thus, fluorescent experiments showed that EAUC exhibited high selectivity and sensitivity with a limit of a real-time H_2_S detection of 23.53 μM. The detection of H_2_S by EAUC was based on the introduction of active metal centre (Ag^+^, H_2_S-responding site) to the Eu^3+^@UiO-66-(COOH)_2_ as the lanthanide-luminescence sensitiser.^96^ MTT assay and cell viability analysis in PC12 cells demonstrated relatively low cytotoxicity for EAUC and biocompatibility. Finally, the determination of H_2_S was tested in diluted fetal bovine and human serum samples demonstrating that EAUC can detect H_2_S in real biological samples. Zhang and co-workers^97^ covalently modified (*via* click chemistry) PCN-58 with target-responsive two-photon (TP) organic moieties to fluorescently detect H_2_S. These TP-MOF probes showed good photostability, high selectivity, minor cytotoxicity, and excellent H_2_S sensing performance in live cells. These modified PCN-58 materials also showed intracellular sensing and depth imaging capabilities (penetration depths up to 130 μm).^97^

**Fig. 16:**
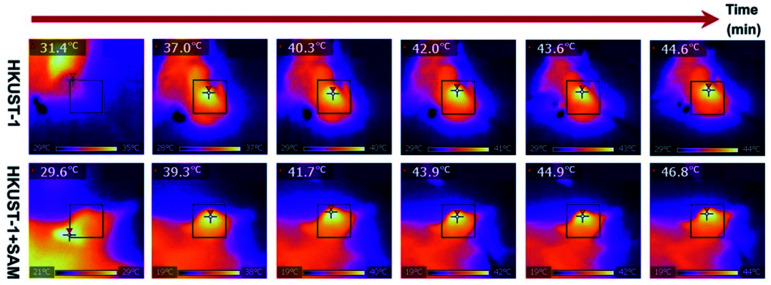
*In vivo* antitumour efficacy of HKUST-1. Photothermal images of the tumour site after 4 h of intratumoural injection of HKUST-1 and HKUST-1 + SAM exposure to laser irradiation (808 nm, 1 W cm^−2^) at the 2 min interval. Reprinted with permission from ref. 98. Copyright (2020) American Chemical Society.

Up to this point we have presented remarkable examples of MOF materials for the detection of H_2_S (biomarkers). All of these have been constructed with the fundamental principle of incorporating into these MOFs “the right chemical functionality” which can react with H_2_S. Remarkably, Wang and Xie^98^ took a step forward and very recently showed how an endogenous MOF-biomarker-triggered “turn-on” strategy was capable to produce therapeutic agents *in situ*, as a promising example in nanomedicine for the precise treatment of colon cancer. HKUST-1 was used as an endogenous H_2_S-activated copper MOF which demonstrated to synergistically mediate H_2_S-activated near-infrared photothermal therapy and chemodynamic therapy. As a proof of principle, the protocol worked as follows: in normal tissues, the photoactivity of as-synthesised HKUST-1 nanoparticles was in the “OFF” state, and no obvious adsorption within the NIR region was observed. On the other hand, when HKUST-1 nanoparticles with high overexpression of H_2_S reached the colon tumour tissues, these nanoparticles were activated to the “ON” state by reacting with endogenous H_2_S to produce *in situ* photoactive copper sulphide with stronger NIR absorption, which is viable for the subsequent photothermal therapy and corresponding thermal imaging (see Fig. 16). In addition, besides the *in situ* formation of the photothermal agent, HKUST-1 nanoparticles also showed a horseradish peroxidase (HRP)-mimicking activity to efficiently convert overexpressed H_2_O_2_ within cancer cells into more toxic ˙OH radicals for chemodynamic therapy. Thus, this promising H_2_S-triggered “turn-on” strategy based on the endogenous biomarker from the tumour microenvironment can provide a precise diagnosis, marginal invasion, and possibly clinical translation.^98^

## 4. Ammonia

NH_3_ is one of the most important chemicals in the world since it is an irreplaceable feedstock for global agriculture and industry (*e.g.*, fertilizers, drug production, heat pumps and fuel cells).^99^ Although fundamental to our diet supply and economy, NH_3_ is a highly toxic (even in small concentrations) and corrosive gas (*i.e.*, difficult to handle and store). Thus, considerably large emissions of NH_3_ from livestock breeding, industrial production, fertiliser application and public transportation can have serious consequences on the environment (highly unfavourable to air quality and aquatic life), and human health.^100^ In addition, NH_3_ reacts with NO_*x*_ and SO_2_ to form PM_2.5_, seriously contributing to the increase of air pollution.

NH_3_ is a colourless gas with a characteristic pungent smell which is typically liquefied and confined in metal tanks in order to be efficiently stored and transported.^101^ This implies high pressures (approximately 18 bar) and constant corrosion of pipelines and containers. Thus, new sorbent technologies capable of efficiently removing or storing NH_3_ are highly desirable for air remediation and separation of NH_3_ from N_2_ and H_2_ since NH_3_ is considered an interesting energy intermediate.^102^ It is clear that capture and recycling of NH_3_ implies a dual connotation in the fields of environment and energy. Classic materials such as activated carbons, zeolites, silicas and even organic polymers have been investigated for the capture and separation of NH_3_ showing low uptakes, poor selectivity and, in some cases, irreversible storages.^103,104^ MOFs have been postulated as a promising option for NH_3_ capture, due to the access to a wide range of chemical functionality (*e.g.*, Lewis or Brønsted acid sites which provide higher affinity to the basic NH_3_ molecule).^104^ However, the main problem that most MOF materials exhibit poor chemical stability towards NH_3_.^105^ The following section describes chemically stable MOFs with interesting NH_3_ capture performances.

### 4.1 MOFs for NH_3_ capture

The NH_3_ molecule is a Lewis and Brønsted base which is the key to its adsorption on the porous surface of chemically stable MOFs. Thus, the incorporation of acidic functional groups within the MOFs is required to achieve relevant NH_3_ capture results. These functionalisations can be organised in three main types: (i) coordinatively unsaturated metal sites (open metal sites), (ii) μ-OH groups and (iii) defective sites.

#### (i) Coordinatively unsaturated metal sites

The majority of MOF materials evaluated for the capture of NH_3_ have demonstrated to be chemically unstable.^106^ However, really interesting examples have been reported by Dincă and co-workers.^107^ They reported a series of new mesoporous MOFs constructed from extended bisbenzenetriazolate ligands and coordinatively unsaturated metal sites (Mn^2+^, Co^2+^, and Ni^2+^), which showed high and reversible NH_3_ uptakes (15.47, 12.00, and 12.02 mmol g^−1^, respectively).^107^ During desorption at different temperatures, all three materials showed pronounced hysteresis which emphasised the strong interaction between the open metal sites and NH_3_. Such bound NH_3_ molecules can be fully removed upon heating the materials up to 200 °C under dynamic vacuum. Interestingly, none of the three materials showed a decrease in the NH_3_ uptake capacity upon cycling. Thus, the high chemical stability of azolate MOFs with open metal sites, towards NH_3_ postulate them as promising alternatives in environmental applications such as the capture of corrosive gases from power plant flue gas streams. This chemical stability can be attributed to the strength of the N atom coordinated to divalent metal centres as described previously.^21^ In this case, the use of linear bistriazolate ligand, in combination with late transition metals such as Ni^2+^, increases the heterolytic metal–ligand bond strength due to its higher basicity (compared to a carboxylate ligand) which allows better MOF stability to acidic gas exposure. This trend is also explained by Pearson's acid and base principle due to Mn^2+^, Cu^2+^ and Ni^2+^ are considered soft cations due to their charge/radius ratio, and nitrogen-based linkers are softer than carboxylate linkers.^35^

**Fig. 17:**
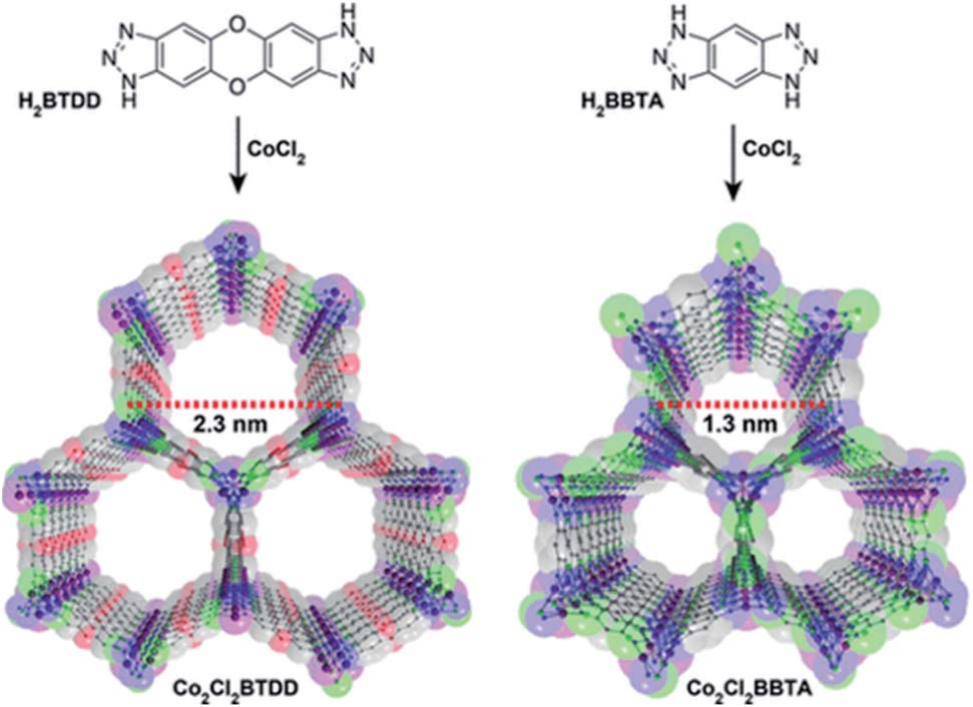
Structure of Co_2_Cl_2_ BTDD (left) and Co_2_Cl_2_ BBTA (right). C, gray; O, red; N, blue; Cl, green; Co, purple. Hydrogen atoms have been omitted for clarity. Reprinted with permission from ref. 108. Copyright (2018) American Chemical Society.

Later, the Dincă's group reported another series of microporous triazolate MOFs (containing open metal sites; Cu^2+^, Co^2+^, and Ni^2+^, see Fig. 17), that exhibited remarkable static and dynamic NH_3_ capacities (up to 19.79 mmol g^−1^, at 1 bar and 298 K).^108^ These isoreticular analogues to the bisbenzenetriazolate examples,^107^ are constructed from smaller triazolate ligands and therefore, they exhibited smaller pore windows (see Fig. 17). Interestingly, these microporous MOFs showed higher NH_3_ captures than their mesoporous analogues.^107^ This remarkable property was not only accounted by the increase in the density of open metal sites, in addition cooperative proximity effects resulted very important. NH_3_ breakthrough experiments on these microporous materials showed the potential applicability for both personal protection and gas separations (NH_3_ capacities of 8.56 mmol g^−1^). Finally, once again, the superior chemical stability of these triazolate MOFs arises, as previously described, from the coordination of the N atom to metal centres (*vide supra*).

**Fig. 18:**
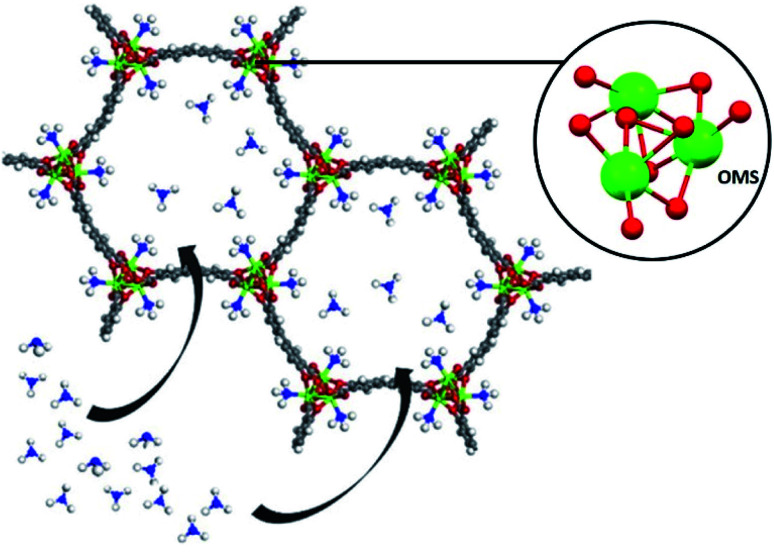
Atomic structure of M_2_(dobpdc) complexes with one-dimensional hexagonal channels. It is possible to observe that each coordinatively unsaturated metal site points towards the channel Reprinted (adapted) with permission from ref. 109. Copyright (2020) John Wiley & Sons.

Very recently, Hong and co-workers recently presented the NH_3_ adsorption properties of M_2_(dobpdc) MOFs (M = Mg^2+^, Mn^2+^, Co^2+^, Ni^2+^, and Zn^2+^; dobpdc^4−^ = 4,4-dioxidobiphenyl-3,3-dicarboxylate), which after activation exhibited open metal sites (see Fig. 18).^109^ The NH_3_ uptake of Mg_2_(dobpdc) at 298 K and 1 bar was 23.9 mmol g^−1^ at 1 bar and 8.25 mmol g^−1^ at 570 ppm, representing the NH_3_ record high capacities at both pressures among existing porous adsorbents.^109^ Mg_2_(dobpdc) remarkably demonstrated chemical stability to not only dry NH_3_, but also to wet NH_3_ (NH_3_·H_2_O vapour). Three consecutive NH_3_ sorption isotherms (activation included only application of vacuum) demonstrated the cyclability of Mg_2_(dobpdc). To evaluate the affinity of the NH_3_ molecule to the open Mg^2+^ sites in Mg_2_(dobpdc), NH_3_-TPD curves demonstrated (based on the Redhead analysis) the activation energy for NH_3_ desorption of 146.7 kJ mol^−1^. The NH_3_ adsorption mechanism of Mg_2_(dobpdc) was investigated by *in situ* FTIR experiments, indicating that NH_3_ was preferentially adsorbed, by coordination bonds, to the open metal sites of Mg_2_(dobpdc). As the MOF sample was exposed to atmosphere the adsorbed NH_3_ in Mg_2_(dobpdc) was converted to NH_4_^+^, due to its reaction with atmospheric moisture. Dynamic NH_3_ breakthrough curves under dry conditions (0% relative humidity (RH) and 298 K) showed a NH_3_ capacity of 8.37 mmol g^−1^ for Mg_2_(dobpdc). When performing these NH_3_ breakthrough experiments under 80% RH conditions, the NH_3_ capacity of Mg_2_(dobpdc) decreased to 6.14 mmol g^−1^, due to the competitive adsorption between NH_3_ and H_2_O. Additionally, this NH_3_ capacity remained constant over five breakthrough wet cycles.^109^ Thus, Mg_2_(dobpdc) is an exceptional MOF material that can be easily synthesised (*i.e.*, microwave-assisted), highly recyclable (even after exposure to a humid NH_3_) without structural degradation or NH_3_ capacity loss and holds the record for the highest NH_3_ uptake at room temperature and atmospheric pressure.

All of these relevant properties are possible due to the exceptional chemical stability of this Mg^2+^-based MOF material. But why is it so stable towards NH_3_? The authors proposed that such chemical stability was due to the higher affinity of Mg^2+^ to oxygen atoms than nitrogen atoms, as confirmed by van der Waals (vdW)-corrected density functional theory (DFT) calculations. They further investigated the origin of such remarkable chemical stability finding that the oxygen adjacent to the carboxylate participates in the coordination of the ligand to the Mg^2+^ cation acting as a tetratopic linker, similar to a chelate effect, increasing the stability of the cluster. This strategy is widely used to prevent pore collapse due to the activation energy barrier to ligand reorganisation or removal, which is increased by the higher linker connectivity.^21^

#### (ii) μ-OH groups

**Fig. 19:**
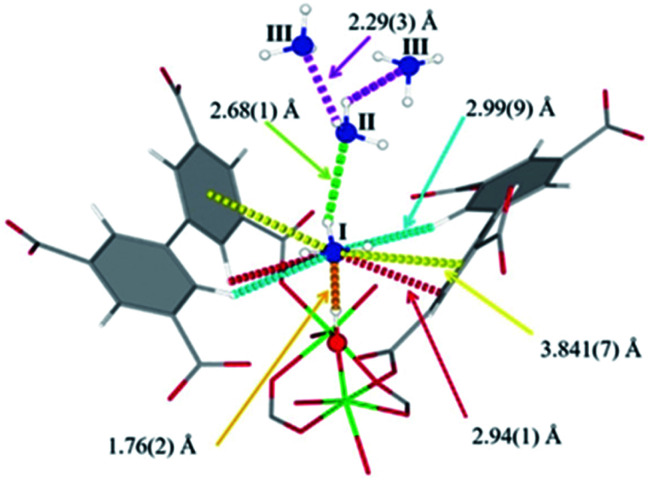
Binding sites for ND_3_ molecules adsorbed within MFM-300(Al) determined by *in situ* NPD studies. Reprinted with permission from ref. 110. Copyright (2018) John Wiley & Sons.

The MFM-300(M) family (see the section of SO_2_) demonstrated remarkable NH_3_ adsorption properties with high uptakes and attractive cyclabilities. First, MFM-300(Al), reported by Yang and Schröder, showed a total NH_3_ capture of 15.7 mmol g^−1^ at 273 K and 1.0 bar.^110^ Although the NH_3_ capture was not particularly high (even at a lower temperature than typically 298 K), the identification of the preferential NH_3_ adsorption sites was achieved by sophisticated *in situ* neutron powder diffraction (NPD) and synchrotron FTIR microspectroscopy. Thus, a structural analysis *via* Rietveld refinement of NPD data for 1.5 ND_3_ (deuterated ammonia)/Al-loaded MFM-300(Al) identified three distinct binding sites (I, II and III) in [Al_2_(OH)_2_(L)](ND_3_)_3_, (H_4_L = 1,1′-biphenyl-3,3′,5,5′-tetracarboxylic acid = C_16_O_8_H_6_) (see Fig. 19). This cooperative network of ND_3_ molecules spreads down the length of the 1D channel, fixed in place by site I. Bond distances for sites II⋯III and a slightly lengthened site I⋯II are similar to a characteristic inter-molecular bond between ND_3_ molecules in the solid state at very low temperature (*i.e.*, 2 K (N⋯D = 2.357(2) Å)), while the bond between the framework μ_2_-OH and site I is considerably shorter. Upon increasing the ND_3_ loading (from 0.5 ND_3_/Al to 1.5 ND_3_/Al), a general shortening of the framework μ_2_-OH⋯site I and sites I⋯II and an increase in the site II⋯III was observed.

Refinement of the NPD data for ND_3_-loaded MFM-300(Al) demonstrated that on increasing the loading and thus the ND_3_/μ_2_-OH ratio, the hydrogen atoms of the hydroxo functional groups experienced a reversible site exchange with the deuterium from the guest ND_3_ molecules residing at site I in the pore to resulting in the formation of μ_2_-OD moieties.^110^ Interestingly, this H–D reversible exchange did not lead to any detectable structural degradation of the long-range order of the MOF material. Thus, the adsorption of ND_3_ in MFM-300(Al) showed a new type of adsorption mechanism where adsorbent and adsorbate experienced a rapid site-exchange *via* reversible formation and cleavage of O–H and O–D chemical bonds, in other words a pseudo-chemisorption binding mechanism.

Very recently, Yang and Schröder expanded the investigation on more MFM-300(M) (M = Fe^2+^, V^4+^, Cr^3+^, In^3+^) materials and NH_3_.^111^ MFM-300(M) (M = Fe^2+^, V^3+^, Cr^3+^) demonstrated fully reversible NH_3_ capture for over 20 adsorption–desorption cycles, under pressure-swing conditions, reaching capacities of 16.1, 15.6, and 14.0 mmol g^−1^, respectively, at 273 K and 1 bar. In the case of MFM-300(In), a significant loss of NH_3_ capture capacity over repeated NH_3_ cycles was shown which corroborated its chemical instability. On the other hand, MFM-300(V^4+^) exhibited the highest NH_3_ uptake (17.3 mmol g^−1^) among the MFM-300(M) family. Interestingly, the NH_3_ desorption phase for MFM-300(V^4+^), with pore dimensions of approximately 6.7 × 6.7 Å^2^, showed a hysteresis loop. Although this could indicate a characteristic capillary NH_3_ condensation (*e.g.*, in mesopores and/or due to a broad distribution of pore size and shape), the authors demonstrated, taking into account the pore dimensions of this MOF, a specific and potentially strong host–guest charge transfer upon the adsorption of NH_3_. In addition, MFM-300(V^4+^) showed an increase of both NH_3_ capacity and residue within the first 18 cycles, which was not observed for the rest of the MFM-300(M) materials. Such residual amount of NH_3_ left within MFM-300(V^4+^) upon regeneration (pressure-swing) gradually increased from 8 to 20% along these cycles, indicating an increase of strongly bound NH-derived species in MFM-300(V^4+^). This NH-derived residue, which was not desorbed by only reducing the pressure, was completely removed by increasing the temperature under dynamic vacuum, although some structural degradation of MFM-300(V^4+^) was observed. The chemical stability of these materials to NH_3_ under humid conditions was investigated by PXRD, confirming the retention of their crystallinity for both MFM-300(M) (M = Cr^3+^, V^3+^), while MFM-300(M) (M = Fe^3+^, V^4+^) showed some structural degradation.^111^

**Fig. 20:**
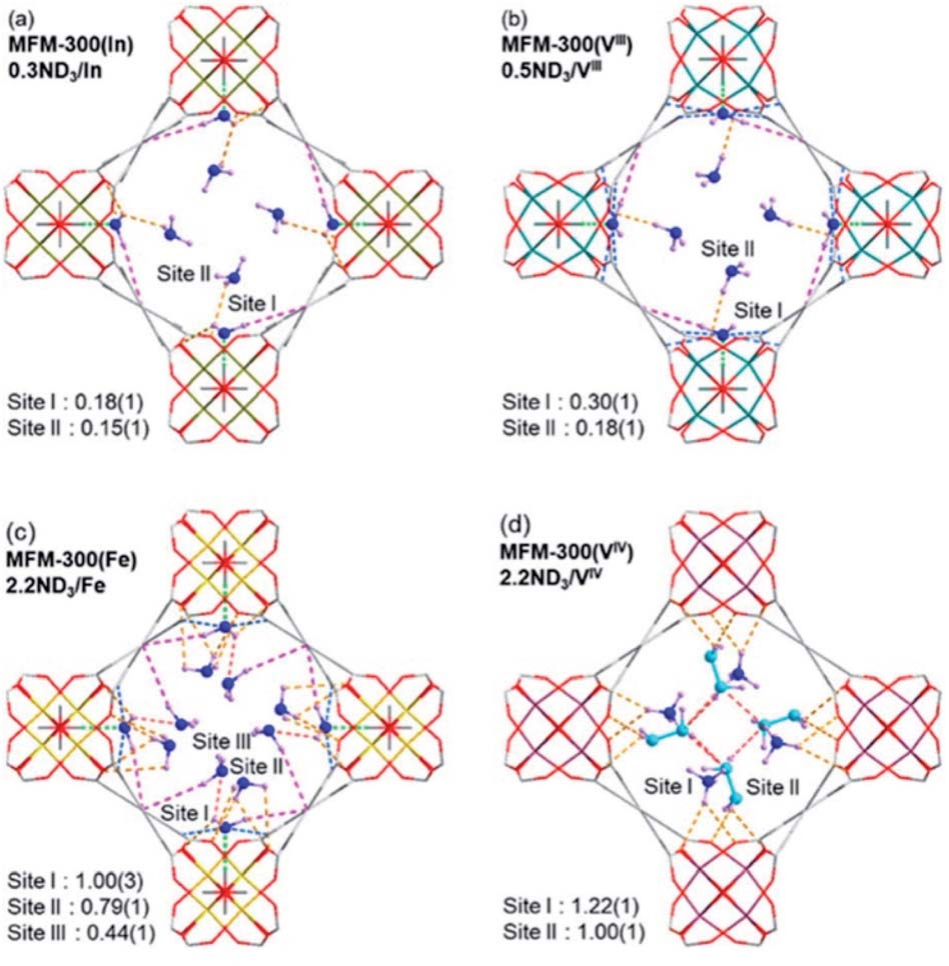
Preferential ND_3_ binding sites in MFM-300(M) family (M = Cr, Fe, V^3+^, V^4+^) determined by NPD at 10 K. Views along *c* axis. Reprinted with permission from ref. 111. Copyright (2021) American Chemical Society.

Neutron powder diffraction data for ND_3_-loaded MFM-300(M) combined with Rietveld refinements, showed the preferential binding sites for ND_3_ (Fig. 20). Two binding sites for ND_3_ were clearly identified for MFM-300(M) (M = In^3+^, V^3+^), while MFM-300(Fe) has an additional binding site for ND_3_. Site I showed the highest occupancy, with hydrogen bonding between the hydroxo functional group and the ND_3_ molecule (μ_2_-OH⋯ND_3_) (1.411–1.978 Å), complemented by additional hydrogen bonding from the organic ligand (H_aromatic_⋯ND_3_ = 2.738–3.174 Å; ND⋯O_ligand_ = 3.078–3.179 Å) and electrostatic interactions (ND_3_⋯aromatic rings = 2.946–3.132 Å) (Fig. 20). Similar to MFM-300(Al),^110^ hydrogen/deuterium site exchange was also observed between the adsorbed ND_3_ and the μ_2_-OH group on for MFM-300(M) (M = In^3+^, Fe^3+^, V^3+^). Site II is located toward the centre of the pore (fixed by hydrogen-bonding interactions ND_3_⋯O_ligand_ = 2.284–3.065 Å). Site III in MFM-300(Fe) is constructed by electrostatic interactions (ND_3_⋯aromatic rings = 3.146 Å). Additionally, in MFM-300(Fe), intermolecular hydrogen bonds between ND_3_ molecules (2.327 Å) were identified, propagating along the 1D channel to form a cooperative {ND_3_}_∞_ network.

The preferential ND_3_ binding sites for MFM-300(V^4+^) were identified and these were different to the previous examples. MFM-300(V^4+^) is not constructed with μ_2_-OH groups. It shows bridging oxo centres to balance the charge of the oxidised V centre. Thus, ND_3_ molecules are located in the centre of the pore. This particular arrangement implicates a very interesting situation: chemical reaction between the ND_3_ molecules: a N_2_D_4_ molecule was formed and located at site II with a ND_3_ molecule at site I (being partially protonated to ND_4_^+^). Then, both sites are stabilised *via* hydrogen bonding (ND_3_⋯O_ligand_ = 2.529–3.092 Å), and the amount of N_2_D_4_ at site II was estimated as 0.5 N_2_H_4_ per V centre.

The formation of N_2_H_4_ was explained in terms of the redox activity of MFM-300(V^4+^) which promotes a host–guest charge transfer between the material and the adsorbed NH_3_ molecules, promoting the reduction of the V^4+^ centres and oxidation of NH_3_ to N_2_H_4_.^111^ These host–guest charge transfer results were experimentally determined by EPR and corroborated by bond valence sum (BVS) calculations, finding as well that the charge transfer between adsorbed NH_3_ molecules and the V^4+^ centre can only occur when the loading of NH_3_ is sufficiently high so that a predominant occupancy of the N site, which is located close to the metal chain, is reached to initiate the redox reaction. Finally, such mechanisms could also be investigated by the approach of metal–ligand dynamics as recently proposed by Brozek and co-workers,^73^ to fully understand the interaction of NH_3_ and the hydroxo functional group in the MFM-300(M) family.

#### (iii) Defective sites

As previously described in the SO_2_ capture section (*vide supra*), crystal irregularities can provide very particular physical and chemical properties. Then, defects in MOF materials also demonstrated remarkable NH_3_ adsorption properties. Very recently, Wu and Tsang presented a comprehensive study on the responsive adsorption behaviours of defect-rich Zr-based MOFs upon the progressive incorporation of NH_3_.^112^ They investigated UiO-67 and UiO-bpydc containing 4,4′-biphenyl dicarboxylate and 4,4’-(2,2′-bipyridine) dicarboxylate ligands, that despite their structural similarities demonstrated a drastic difference in the NH_3_ adsorption properties when the biphenyl groups in the organic ligand were replaced by the bipyridine moieties. Such replacement can confer flexibility to the framework in the context of mainly ligand “flipping” but without significant pore volume alteration.

**Fig. 21:**
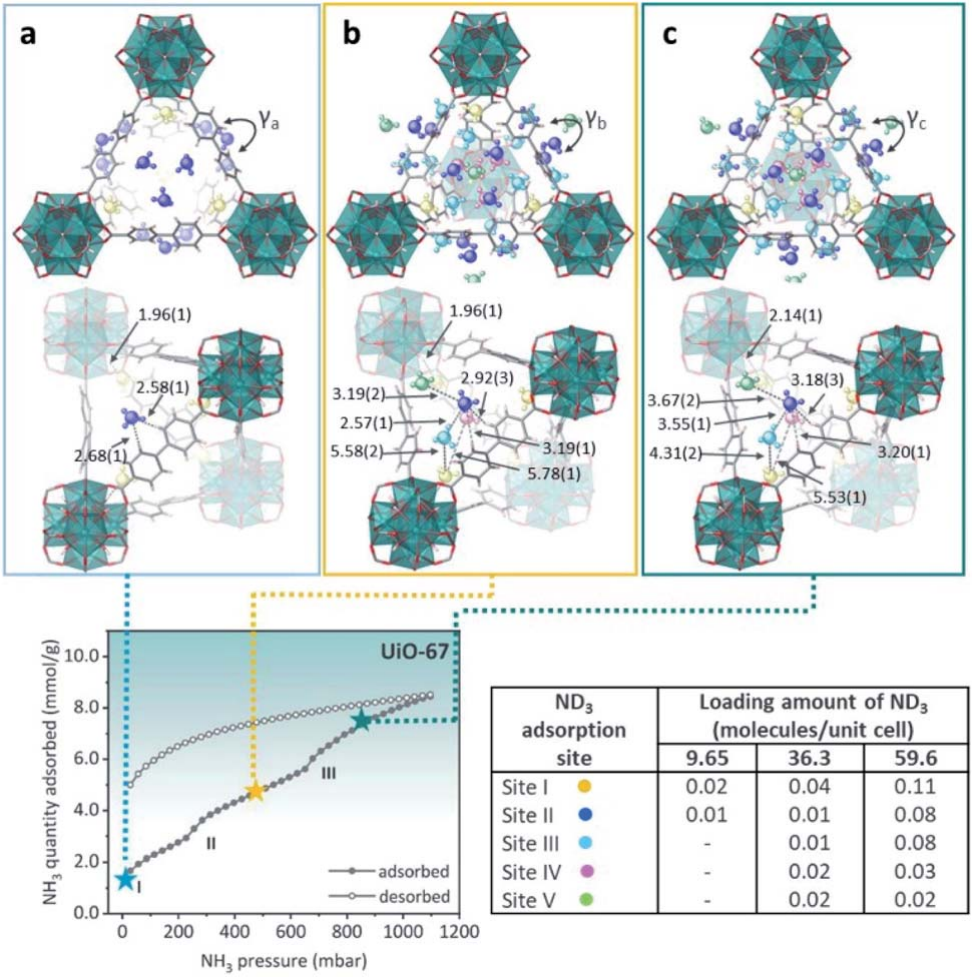
(Top) Refined ND_3_ positions in the UiO-67 structure at ND_3_ loadings of (a) 1.23, (b) 4.63, and (c) 7.60 mmol g^−1^ obtained from NPD data at 300 K. ND_3_ molecules at different binding sites: site I: yellow, site II: blue, site III: light blue, site IV: pink, and site V: green. (Bottom) NH_3_ sorption isotherm at 298 K and up to 1100 mbar; adsorption: closed circles; desorption: open circles reprinted with permission from ref. 112. Copyright (2021) American Chemical Society.

Then, defective UiO-67 (non-monodisperse pore structure created by missing ligand defects) was synthesised thanks to the high connectivity of the Zr_6_ oxoclusters, which help to retain the overall crystal structures even with some ligands are missing. UiO-67 involves uniform trigonal windows with a diameter of 11 Å that lead to interconnected tetrahedral and octahedral pores inside the structures. In the presence of missing ligand defects, the trigonal windows surrounding the defects in UiO-bpydc are fused into lozenge windows with a dynamic size larger than 14 Å. NH_3_ adsorption–desorption isotherms at 298 K and up to 1 bar were performed for both materials: UiO-67 and UiO-bpydc. In the case of Ui-O-67, the adsorption isotherm showed “step-like shape” with two events (see Fig. 21, bottom). This characteristic isotherm shape is caused in MOFs by a gate-opening phenomenon due to the interaction between guest NH_3_ molecules and pore walls, or a pore filling process.^113^ Thus, at the beginning of the adsorption, the rapid and sudden increase in NH_3_ adsorption at approximately 30 mbar (1.70 mmol g^−1^) (position I) suggests the presence of strong adsorption sites inside the framework, *e.g.*, strong interaction of the NH_3_ molecules with the hydroxo (μ_3_-OH) functional groups. Later, the NH_3_ uptake increased from 2.40 to 4.40 mmol g^−1^ at 250 mbar (position II) and from 5.60 to 8.40 mmol g^−1^ at 650 mbar (position III, Fig. 21). The desorption phase exhibited large, opened hysteresis, indicating that the NH_3_ molecules strongly interact with the pore walls of the material.^112^ These NH_3_ molecules were fully desorbed by heating up to 423 K for 1 h under dynamic vacuum. Later, the chemical stability of UiO-67 towards NH_3_ was investigated by conducting three NH_3_ adsorption–desorption cycles and finally exposing the material to NH_3_ vapour for 1 week. PXRD experiments corroborated the stability of UiO-67 to dry and humid NH_3_. Then, in order to identify the preferential NH_3_ adsorption sites in UiO-67, *in situ* high-resolution neutron powder diffraction (NPD) and synchrotron powder X-ray diffraction (SPXRD) experiments were carried out. The refined structures of UiO-67 at different ND_3_ pressures, reviled five such adsorption preferential sites (Fig. 21).^112^ At site I, the ND_3_ was found close to the μ_3_-OH with a OH⋯N_I_ distance of 1.96(1) Å, and O⋯N_I_ distance of 2.80(1) Å (see Fig. 21). This distance suggested the formation of a relatively strong H-bonding interaction between the μ_3_-OH and ND_3_, similar to the one reported by Yang and Schröder in the MFM-300(M) family.^110,111^ ND_3_ molecules at site II were situated close to the walls of the trigonal windows suggesting interactions with the MOF organic ligands (H_ligand_⋯N_II_ = 2.58(1) and 2.68(1) Å), Fig. 21. On increasing the amount of ND_3_, these molecules were located near to the tetragonal pore (sites III and IV). At site III, the ND_3_ molecule filled the shallow pore positions with N_II_⋯N_III_ bond distances among these ND_3_ sites in the range of 2.33(1) and 2.57(1) Å, creating a H-bonded network of these ND_3_ molecules (Fig. 21).

**Fig. 22:**
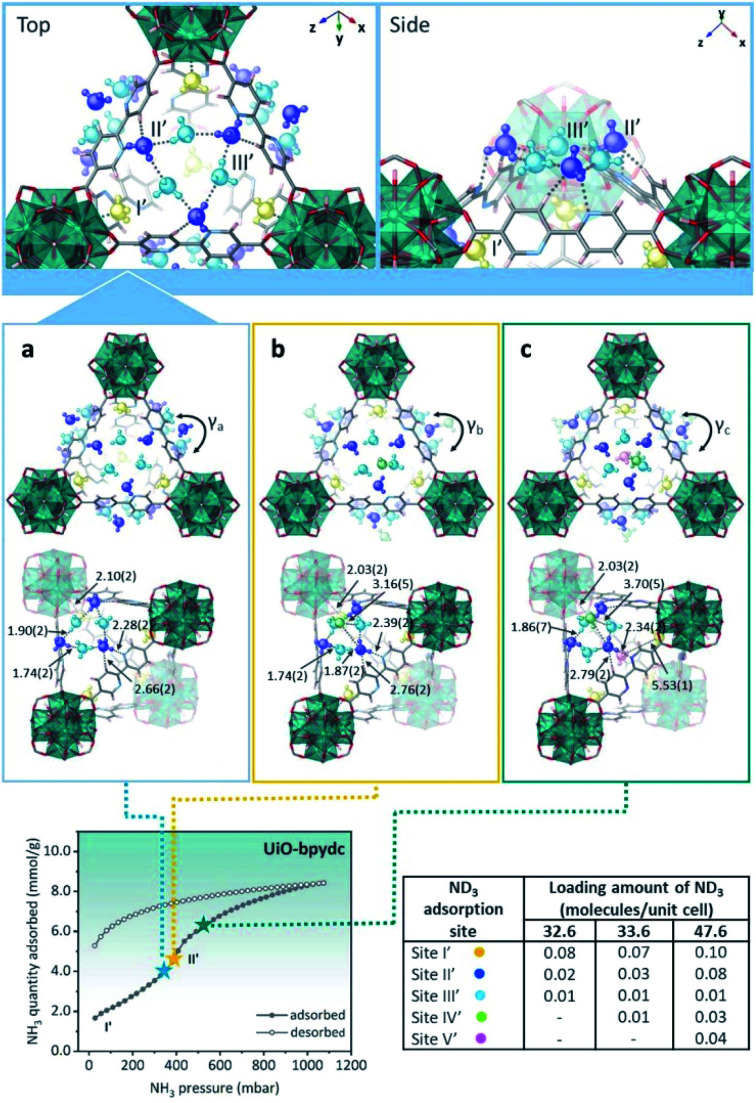
(Top) Refined ND_3_ positions in the UiO-bpydc structure at ND_3_ loadings of (a) 4.15, (b) 4.27, and (c) 6.06 mmol g^−1^ obtained from NPD data at 300 K. (Bottom) ND_3_ molecules at different binding sites: site I: yellow, site II: blue, site III: light blue, site IV: green, and site V: pink. NH_3_ sorption isotherm at 298 K and up to 1100 mbar; adsorption: closed circles; desorption: open circles reprinted with permission from ref. 112. Copyright (2021) American Chemical Society.

In the case of UiO-bpydc, the NH_3_ adsorption isotherm exhibited an uptake of 8.4 mmol g^−1^ at 298 K and 1 bar (see Fig. 22). Conversely to UiO-67, UiO-bpydc showed only one large and sharper transition step (position II′) for the NH_3_ adsorption phase (Fig. 22). Then, *in situ* NPD experiments and followed by Rietveld refinements corroborated the existence of missing ligand defects. Similarly to UiO-67, the preferential NH_3_ adsorption sites within UiO-bpydc were also identified by *in situ* NPD experiments (replacing NH_3_ by ND_3_). Thus, two independent preferential sites of ND_3_ were found close to the μ_3_-OH functional group (site I′) and to the organic ligands (site II′, bipyridine ligand), Fig. 22. This strongly suggested that the adsorption of ND_3_ mainly takes place at site II′. For site I′, the OH⋯N_I_ bond distance was estimated to be 2.10(2) Å, which is similar to that found in the isostructural UiO-67. Interestingly, the ND_3_–bipyridine ligand interaction was estimated to be stronger than the ND_3_–biphenyl ligand interaction with longer distances (H_ligand_⋯N_II_ = 2.68(1), 3.19(1), and 3.20(1) Å, respectively) (see Fig. 22). Increasing the dosing of ND_3_ molecules, increased the H-bonding between the ND_3_ molecules giving rise to a network of six ND_3_ molecules with the three bipyridine ligand located around the trigonal window. This particular situation demonstrated the gate-opening/closing behaviour *via* ligand flipping of UiO-bpydc upon ND_3_ adsorption.^112^

This phenomenon was further investigated by DFT calculations to understand the role of the bipyridine ligands of UiO-bpydc in forming the H-bonding network with NH_3_ molecules, in comparison with the biphenyl ligands of UiO-67. Thus, the DFT-optimised structures demonstrated the progressive distortion of the aligned bipyridine ligands with *γ* changed from 20.34° (in good agreement with the NPD refined desolvated structure of UiO-bpydc) to 19.47°, 10.83°, 9.49°, and 6.33° by increasing the number of NH3 molecules at the trigonal window. Finally, these remarkable results demonstrated that the different pore openings (windows) induced by missing ligands can introduce stepped NH_3_ sorption with a strong hysteresis into the UiO type MOFs (*i.e.*, biphenyl ligands are replaced by pyridine ligands).^112^

## 5. NO_*x*_

Nitrogen oxides (NO_*x*_ = N_2_O, NO, N_2_O_3_, NO_2_ N_2_O_4_ and N_2_O_5_) are considered one of the major air pollutants generated by anthropogenic activities, particularly those that involve fuel combustion from stationary and mobile sources such as thermal power plants and vehicles.^114^ It has been estimated that fossil fuel combustion generates around 5% of NO_2_, and 95% NO.^114^ Nevertheless, once NO is released into the atmosphere, it rapidly reacts with O_2_ to form NO_2_. The latter is a poisonous red-orange gas responsible for the reddish-brown colour of the smog, although at lower temperatures it can dimerize into a colourless N_2_O_4_ dimer. Due to the highly reactive nature of NO_*x*_, the uncontrolled emissions of such gases into the atmosphere are associated with a series of health and environmental issues.^114–116^ For instance, the presence of high concentrations of NO_2_ has been directly related to the formation of tropospheric ozone O_3_,^109,116^ which not only is considered an important greenhouse gas; but is also associated with pulmonary and chronic respiratory diseases.^115,116^ The adverse effects caused by atmospheric NO_2_ have motivated an intense research for the development of NO_2_ abatement technologies.^114,117^

One of the most studied methods to mitigate the atmospheric NO_2_ is the development of porous materials for the selective capture of this pollutant. The traditional adsorbent materials used for NO_2_ removal include zeolites,^118^ calcium-based adsorbents,^119^ and activated carbon.^120^ However, such materials are typically affected by the reactive oxidative nature of NO_2_, which hampers the fully reversible desorption of the guest molecules and limits the material regeneration.^117^

### 5.1 MOFs for NO_*x*_ capture

Current investigations in NO_*x*_ sorption have pointed out that MOFs represent an attractive alternative for storage, selective separation, and/or catalytic transformation of NO_2_.^121^ Such materials not only display a large surface area, but they also exhibit tuneable pore functionalities^122^ allowing for the stabilisation of guest molecules through the formation of supramolecular host–guest interactions. For instance, a study reported by Peterson *et al.*^123^ points out the key role of the organic linker for the capture of NO_2_. In this work, the authors conducted a comparative study bout the NO_2_ sorption performance of UiO-66 and UiO-66-NH_2_.

The gas sorption experiments were performed under dry and controlled humid conditions. The results obtained from the microbreakthrough experiments reveal that UiO-66-NH_2_ exhibits a higher capacity for the NO_2_ sorption than UiO-66 (20.3 mmol g^−1^*vs.* 8.8 mmol g^−1^; respectively). Under humid conditions (80% RH), UiO-66-NH_2_ exhibits higher uptake of NO_2_ than under dry conditions (31.2 mmol g^−1^*vs.* 20.3 mmol g^−1^; respectively), and it produces a significantly lower amount of NO as a by-product than its analogous UiO-66 (4.5%, 9.5%; respectively).

**Fig. 23:**
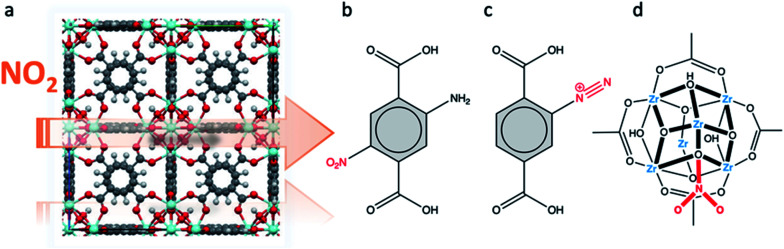
(a) Structure of UiO-66 analogues. Post functionalization of phenyl ring modifications upon NO_2_ sorption: (b) nitration of the phenyl ring, (c) diazonium ion formation. (d) Nitration of the bridging hydroxyl group at the SBU. Reprinted (adapted) with permission from ref. 123. Copyright (2016) John Wiley & Sons.

Such differences were explained in terms of the higher capability of UiO-66-NH_2_ to adsorb H_2_O vapours. As the H_2_O co-adsorbed within the pore network might enhance the stabilization of NO_2_ molecules through the formation of supramolecular interactions and it facilitates the preferential formation of nitrous acid as a by-product. The complementary characterization of UiO-66-NH_2_ upon NO_2_ adsorption revealed that although the crystallinity of UiO-66-NH_2_ remains intact, the high reactivity of the adsorbate leads to a series of reactions with the organic linker, which ends up in the post functionalization of the phenyl group (Fig. 23).^123^

**Fig. 24:**
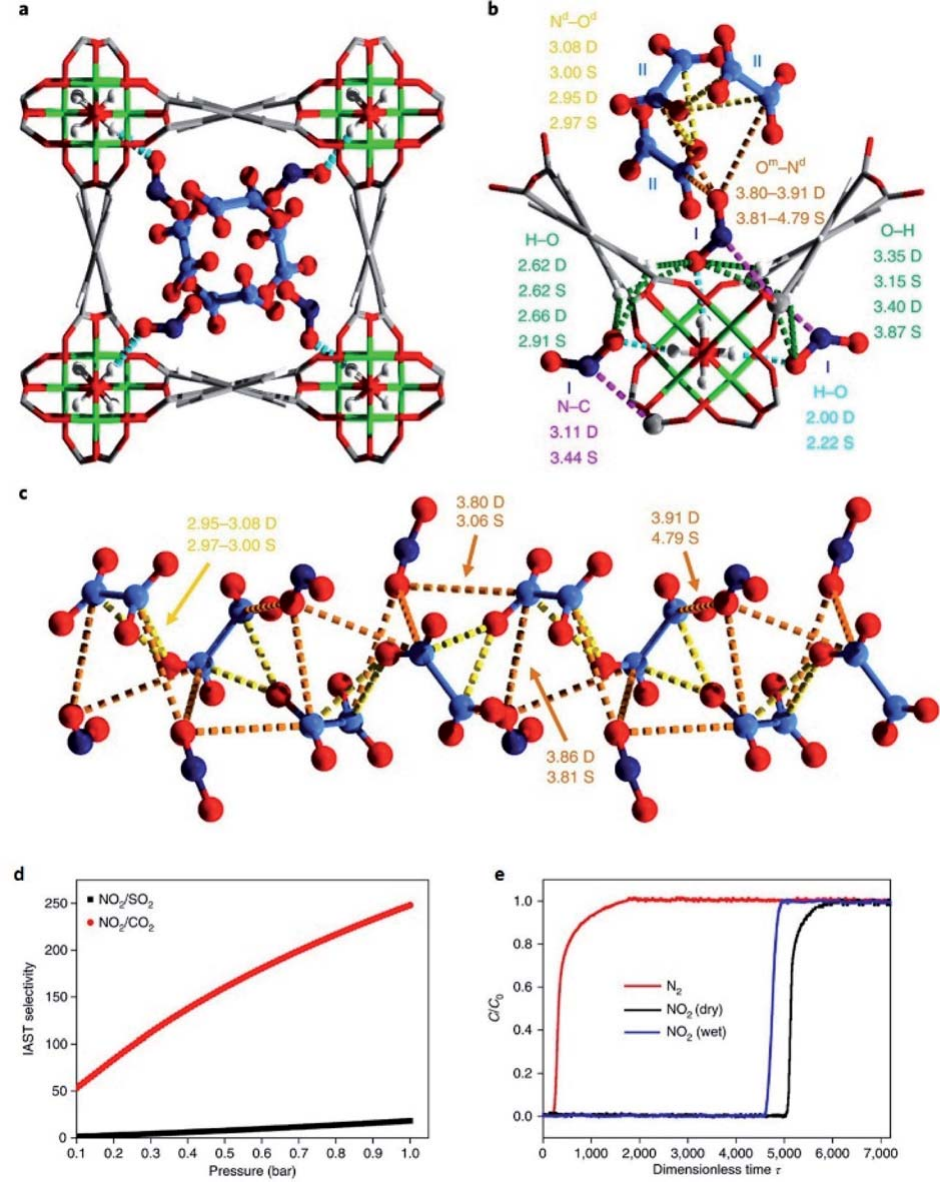
Views of the structural model of MFM-300(Al)·(NO_2_)(N_2_O_4_)_2_. Al-green, C-grey, O-red, H-white, N-blue. (a) and (b) host–guest supramolecular interactions. (c) Structural view of 1D helical chain (NO_2_, N_2_O_4_)_∞_ within the channel of MFM-300(Al). (d) Comparison of IAST selectivities for equimolar mixtures of NO_2_/SO_2_ and NO_2_/CO_2_ at 0.1–1.0 bar for MFM-300(Al) at 298 K. (e) Dimensionless breakthrough curve of 0.5% NO_2_ (5000 ppm) diluted in He/N_2_ under both dry and wet conditions through a fixed bed packed with MFM-300(Al) at 298 K and 1 bar. Reprinted (adapted) with permission from ref. 124. Copyright (2018) Springer Nature.

The main transformation that suffered the organic linker upon NO_2_ uptake were the nitration of the aromatic ring and the formation of the diazonium ion at the amino group, while in the inorganic SBU the terminal –OH group is replaced by the NO^3−^ ion. More recently, a study conducted by Schröder and Yang,^124^ demonstrated that the synergistic effect between the organic ligand and the inorganic building block in MFM-300(Al) allows for the stabilisation of highly reactive NO_2_ species within the pore network (Fig. 24a–c). The authors reported that under ambient conditions MFM-300(Al) exhibits a fully reversible NO_2_ isotherm uptake of 14.1 mmol g^−1^ at 298 K. Moreover, the host material retains its crystallinity and sorption capacity after five cycles of NO_2_ adsorption/desorption. Remarkably, MFM-300(Al) displays outstanding performance for the selective removal of low-concentration of NO_2_ (5000 to <1 ppm) from gas mixtures (Fig. 24d). The optimal uptake and selectivity of MFM-300(Al) for NO_2_ was attributed to the existence of both host–guest and guest–guest interactions. The former involves mainly the hydrogen bonding interaction between NO_*x*_ and the –OH pendant group of the inorganic node, while the latter refers to the supramolecular interactions between the guest molecules in their monomeric (NO_2_) and dimeric (N_2_O_4_) form. Such interactions give rise to a one-dimensional helical chain arrangement comprised of alternating monomer-dimer molecules (NO_2_·N_2_O_4_)^∞^ running along the channel of MFM-300(Al) (Fig. 24d).

**Fig. 25:**
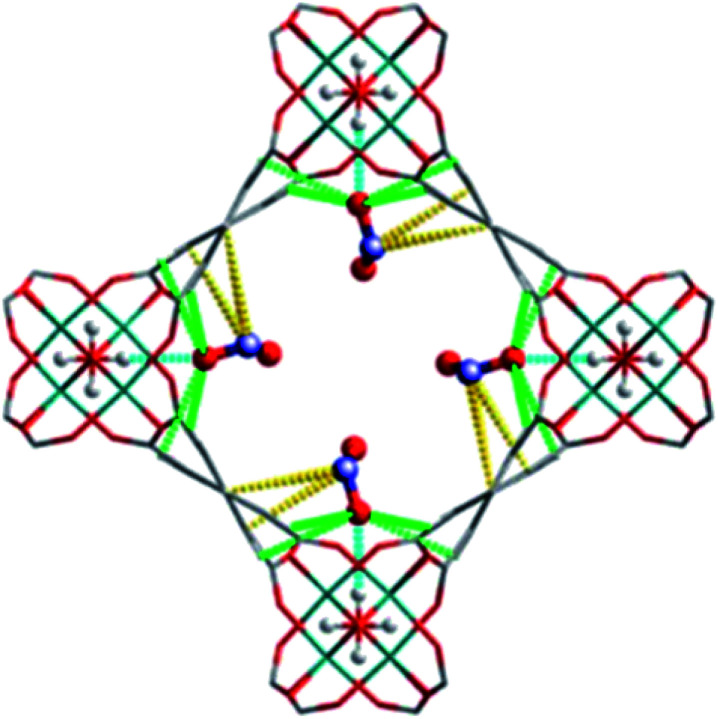
Rapid scan RS model showing the packing of guest molecules within MFM-300(V^3+^). Reprinted with permission from ref. 125. Copyright (2020) American Chemical Society.

The cooperative supramolecular interactions between NO_2_ and N_2_O_4_, confined within the pore network, allows for the stabilization of the highly reactive NO_2_ molecules and inhibits the guest–host electron transfer. This affords an unusual, fully reversible desorption of NO_2_ without altering the framework structure of the host material. The structural versatility of MOFs not only permits the stabilization of highly reactive NO_*x*_ species within the pore channels but also opens the possibility for the catalytic conversion of NO_*x*_ into more valuable and/or less abrasive compounds. In this regard, Schröder and Yang reported the adsorption and catalytic transformation of NO_2_ by using a redox-active MOF,^125^ termed MFM-300(V) [V_2_(OH)_2_(C_16_H_6_O_8_)]. This system not only exhibits high adsorption capacity for NO_2_ (13 mmol g^−1^ at 298 K and 1.0 bar), but it also allows for the catalytic reduction of NO_2_ into NO. The structural analysis reveals that upon gas sorption, NO_2_ molecules get primarily anchored to the pore walls through a hydrogen-bonding interaction with the bridging hydroxyl groups of the inorganic nodes. Further stabilization is reached by forming of 8-fold supramolecular interactions with the aromatic ligand (Fig. 25). Then, those interactions give rise to the host–guest charge transfer process and the formation of NO (through the oxidation of the metal centre from V^3+^ to V^4+^), and water, which is produced by the deprotonation of the bridging –OH groups anchored to the SBU.

Recent research conducted by Dincă and co-workers highlights the capacity of M-MFU-4*l* (M = Cu, Zn) MOFs for NO capture and on-demand desorption of guest molecules by a thermal treatment.^126^ In this study, the authors demonstrated that the gas-sorption properties of the host material can be easily adjusted by modifying the cation identity and the oxidation state of the inorganic building block. To this aim, three different systems were selected as host materials (i) Zn_5_Cl_4_(BTDD)_3_; (ii) Zn_3_Cu^II^_2_Cl_4_(BTDD)_3_; and (iii) Zn_3_Cu^I^_2_Cl_2_(BTDD)_3_ (H_2_BTDD = bis(1H-1,2,3-triazolate[4,5-b;4′,5′-i])dibenzo[1,4] dioxin). The first material Zn_5_Cl_4_(BTDD)_3_ (Zn-MFU-4*l*) displayed the lowest capacity for NO sorption (0.51 mmol g^−1^ at 750 torr). The second system, Zn_3_Cu^II^_2_Cl_4_(BTDD)_3_ (Cu^II^-MFU-4*l*), was obtained by substituting two Zn^2+^ atoms from Zn-MFU-4l by Cu^2+^ ions. This subtle modification resulted in a significant increase in the NO sorption capacity (1.43 mmol g^−1^ at 750 torr).

**Fig. 26:**
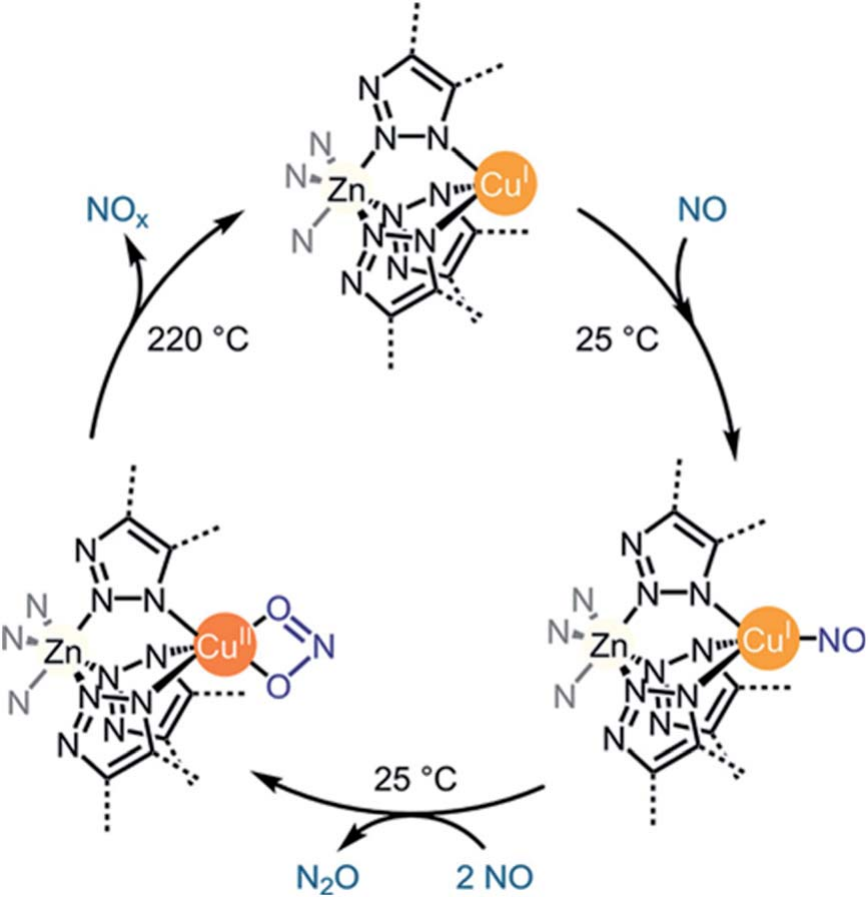
Proposed NO Disproportionation Cycle Using Cu^I^-MFU-4*l*. During the thermal decomposition of Cu-MFU-4*l*(NO_2_), NO is the major NO_*x*_ species detected. Reprinted with permission from ref. 126. Copyright (2021) American Chemical Society.

These findings were explained in terms of the differences in the tetrahedral coordination environment around the metal centre N_3_M–Cl (M = Zn^2+^, Cu^2+^). In the first case, the saturated coordination environment around Zn^2+^ prevents the formation of stronger metal–NO interactions, whereas in the second system, the N_3_Cu–Cl geometry is sufficiently distorted to allow the NO molecules to approach closer to the metal centre leading to stronger Cu–NO interactions. The formation of Cu^2+^–nitrosyl species upon NO adsorption was corroborated by diffuse-reflectance infrared Fourier-transform spectroscopy (DRIFT) study. Finally, the third system (Cu^+^-MFU-4*l*) was obtained by the reduction of Cu^2+^ to Cu^+^ accompanied by the concomitant loss of chloride. This system displays a significant improvement in the low-pressure NO uptake (1.24 mmol g^−1^ below 1.4 torr). This value is three times the amount of NO adsorbed by Cu^2+^-MFU-4*l* (0.0008 mmol g^−1^) and Zn^2+^-MFU-4*l* (0.0002 mmol g^−1^) under the same conditions. The DRIFT analysis suggests that the metal centre in Cu^1+^-MFU-4l allows for the formation of Cu^+^–NO complex even when the material is exposed to low concentrations of NO in Ar (10 ppm). This interaction favours the NO disproportionation 3 NO → NO_2_ + N_2_O and the oxidation of the metal centre to Cu^2+^ which strongly binds to the NO_2_ product (Fig. 26). Then, the Cu^2+^–nitrite bond can be cleaved by exposing Cu^2+^-MFU-4*l*–(NO_2_) to high temperatures, releasing the anchored NO_2_ and thereby regenerating the adsorbent Cu^+^-MFU-4*l*. According to the authors, this catalytic system represents a potentially attractive scheme for cold-start NO capture. The design of porous materials for storage and controlled release of NO have gained relevance in biomedicine, as it has been shown that the exposure to controlled doses of NO induces wound healing and prevents the formation of blood clots. Moreover, NO is a potent antimicrobial agent; therefore, the development of materials for the local release of NO could be highly advantageous to reduce the risk of infections.

One of the first reports about the use of MOFs for the on-demand delivery of NO was published in 2007 by Morris and co-workers.^127^ In this work, the authors used HKUST-1 as a reservoir for NO. The gravimetric adsorption experiments performed at 196 K and 298 K display significant isotherm hysteresis with an adsorption capacity of *ca.* 9 mmol g^−1^ and 3 mmol g^−1^ at one bar, respectively. Upon gas desorption processes, both isotherms exhibit a remaining amount of NO trapped within the pore network (2 mmol g^−1^). The IR analysis of NO-loaded material reveals that the presence of open copper sites in the walls of HKUST-1 framework allows for the formation of NO⋯Cu^2+^ coordination adduct; thereby leading to the irreversible adsorption of NO. However, NO molecules trapped within the pore network can be released on demand upon exposure of the NO-loaded HKUST-1 to water vapours, as H_2_O acts as a nucleophile replacing the NO molecules coordinated to the open copper sites.

More recently, the same research group reported the development of MOF-based composites for the controlled delivery of NO.^128^ The selected host material was CPO-27-Ni, as this MOF affords highly efficient adsorption, storage, and release cyclable profile for NO.^129^ Moreover, this material exhibits high stability towards adsorbed water, which allows for the controlled release of NO when exposed to moisture, without altering the structural arrangement of the host material.

The optimal MOF films were obtained by varying the amount of CPO-27-Ni (wt%) integrated within a polyurethane matrix. The composite films exhibited homogeneous distribution of the MOF within the polymer matrix. The release of NO was triggered by exposing the composite films to 11% or relative humidity. Such conditions allow for the controlled release of the NO adsorbed without affecting the polymer properties. Finally, the antimicrobial properties of the resultant MOF-based composite demonstrated that the controlled release of biologically active levels of NO provides a bactericidal effect against *Escherichia coli* (*E. coli*) and *Staphylococcus aureus* (*S. aureus*).

## 6. Selectivity of trace gases

One important aspect of evaluating MOF materials is their selectivity of adsorbing one gas preferentially over other molecules that are present in the industrial flue gas streams, as these are mainly composed of CO_2_ (10–15% [v]), N_2_ (70–75% [v]), and H_2_O (∼10–18% [v]),^130^ whereas gases such as SO_2_ (0.00325% [v], 500–3000 ppm), H_2_S (13.5% [v]; 1000–4000 ppm), NH_3_ (300–800 ppm) and NO_*x*_ (200–800 ppm) are present in trace concentrations.^131^ For this reason, it is highly desirable that the study of the capture of trace gases by MOFs should be accompanied by a selectivity study in the presence of other gases in order to have a complete profile of their potential applications at industrial levels.

Table 4 lists the most promising MOF materials for the separation of SO_2_ and H_2_S gases, with a good selectivity performance.

**Table 4:** Summary of the adsorption captures of SO_2_ and H_2_S and IAST selectivity of each one in various MOFs materials

Gas target	MOF	Gas adsorption [mmol g^−1^]	Flue gas concentration	Selectivity				Ref.
				CO_2_	N_2_	CH_4_	Other gases	
SO_2_	MIL-160	7.2	SO_2_/CO_2_ (10 : 90–50 : 50 v/v)	124–128 (IAST)	31.25^37*e*^	—	—	18
	MIL-125(Ti)-NH_2_	10.8	SO_2_/CO_2_ (10 : 90–50 : 50 v/v)	42–55 (IAST)	—	—	—	18
	MFM-170	17.5	Equimolar SO_2_/N_2_	35	944	260	203 (CO)	19
	MFM-300(Al)	7.1	50–350 mbar^b^	—	6522	3620	10^5^ (H_2_)	37*a*
							3105(CO)	
							4974 (O_2_)	
	MFM-300(In)	8.28	At 50 : 50 mixture of each gas	60	5000	425	—	37*b*
	MFM-601	12.3	SO_2_/CO_2_, SO_2_/N_2_ (50 : 50 to 10 : 90)	32	255	—	—	132
	ELM-12	2.73	10 : 90 mixture at 298 K and 1 bar	30	4064	871	—	133
	**1@Ba(OH)2**	4.0^a^	N_2_/CO_2_/SO_2_ (83.5 : 14 : 2.5 v/v)	990	—	—	—	45
	SIFSIX-1-Cu	11.01	At 10 : 90 mixture of each gas	70.7	3145.7	1241.4	—	51
	SIFSIX-2-Cu-i	6.9	At 10 : 90 mixture of each gas	87.1	3103.2	1017.1	—	51
	SIFSIX-3-Zn	2.1	At 10 : 90 mixture of each gas	—	506.7	276	—	51
	SIFSIX-3-Ni	2.74	At 10 : 90 mixture of each gas	—	701.8	371.6	—	51
	KAUST-7	1.4^a^	SO_2_/CO_2_/N_2_ (4%:4% : balance) and (500 ppm : 10% : balance)	1	—	—	—	52
	KAUST-8	1.6^a^	SO_2_/CO_2_/N_2_: 0.05/10/89.95	66	—	—	—	52
	ECUT-77	8.0	SO_2_/CO_2_ (1 : 99 v/v; 2000 ppm SO_2_)	44–36	—	—	—	134
	ECUT-100	4.95	At 1 : 99 mixture of each gas	27.5–26.9	—	2302	—	135
	ECUT-111	11.56	SO_2_/CO_2_, SO_2_/N_2_ (1 : 99 v/v)	22.2–25.2	860.9	—	—	136
H_2_S	MIL-101(Cr)	0.419^a^	H_2_S/CO_2_/He (1 : 10 : 89 v/v)	5	—	—	—	63
	HKUST-1	∼1^a^	H_2_S/CO_2_/He (1 : 10 : 89 v/v)	∼40	—	—	—	63
	KAUST-7	—	H_2_S/CO_2_/CH_4_ (20 : 20 : 60)	—	—	20.7^d^	—	139
	KAUST-8	—	H_2_S/CO_2_/CH_4_ (20 : 20 : 60)	—	—	18.6^d^	—	139
	KAUST-8	—	H_2_S/CO_2_/CH_4_ (5 : 5 : 90)	1	—	—	—	137
	SIFSIX-2-Ni-i	—	H_2_S/CO_2_/CH_4_ (5 : 5 : 90)	3.3	—	—	—	137
	MIL-125(Ti)-NH_2_	—	H_2_S 0.001	—	—	70^c^	—	138
	Ga-soc-MOF-1a	4.4	CO_2_/H_2_S/CH_4_ (5% : 5% : 90%)	7	—	—	—	64
	Y-fum-fcu-MOF	1.1^a^	CO_2_/H_2_S/CH_4_ (5% : 5% : 90%)	6.4	—	—	—	139
	[BMIM][Cl]/Cu-TDPAT^e^	—	H_2_S 1000 ppm	—	—	1302	—	140
	Cu-TDPAT^e^	—	H_2_S 1000 ppm	—	—	141	—	140
	MIL-53(Al)^f^	—	CH_4_/CO_2_/H_2_S (60% : 39.9% : 0.1%)	—	—	23–34	—	141
	MIL-47(V)-Br^g^	—	H_2_S/CH_4_ (5 : 95) 0.1 MPa	—	278	68	—	142
	MIL-47(V)^g^	—	H_2_S/CH_4_ (5 : 95) 0.1 MPa	—	179	223	—	142
			H_2_S/CH_4_ (1 : 99) 0.1 MPa			51		
			H_2_S/N_2_ (50 : 50)					

a Breakthrough experimental result.

b Obtained from ratio of slopes of initial adsorption isotherm plot at a low pressure region.

c GCMC simulations at 303 K and 10 bar.

d Membrane composite measured at 35 °C.

e Composite materials.

f Pellets.

g GCMC simulations at 273 K.

## 7. Stability and reusability of MOFs

As previously shown, there is a wide variety of MOFs with high structural stability towards corrosive gases (SO_2_, H_2_S, NH_3_ and NO_*x*_). The stability, as well as the reusability of the MOFs, is required for tangible industrial applications on the adsorption field. Considering that the CSD currently contains near to 70 000 MOF structures,^143^ a selection criterion for MOFs that could be potentially useful in the adsorption of toxic gases is essential, as are the guidelines for generating new materials focused on this target.^144^ Although the nature of MOFs is very diverse, MOFs stable in the presence of corrosive gases feature some common characteristics: (i) the strength of the ligand–metal bond; (ii) oxidation state of the metal centre; (iii) robustness of the metallic cluster; and (iv) the formation of supramolecular host–guest interactions. The presence of at least one of these characteristics is responsible for the stabilization of the framework under such harsh conditions. For example, when the strongest binding site corresponds to a free metal site, the metal–ligand bond should be strong enough to avoid linker displacement and subsequent structure degradation. The combination of ligands with strong donor groups/atoms and late transition metals, or the use of metals with a higher oxidation state together with carboxylate ligands are good synthetic strategies to improve the strength of the metal–ligand bonds and, therefore, increase the overall stability of the framework.^21,40*a*,145^ For example, MOFs formed by triazolate ligands such as the M_2_Cl_2_BTDD family (M = Ni^2+^, Co^2+^, Mn^2+^, and Cu^2+^)^107^ with uncoordinated metal sites are stable even after several sorption and desorption cycles of NH_3_. Such stability increases according to the Irving–Williams series, being the Ni^2+^ framework more stable than the Cu^2+^. In this case, the presence of heterolytic metal–ligand bonds and a less labile metal centre, enhances the MOF's stability. In MFM-170,^19^ a MOF formed by the Cu^2+^ paddlewheel SBU, the coordination of one of the Cu^2+^ centres to a N-donating ligand, allowed reversible SO_2_ adsorption, even if one of the Cu^2+^ atoms presented weak contacts with SO_2_. Additionally, the interactions between SO_2_ and the surface of the framework contributed to the reversibility of the adsorption process. This research represents an example of enhancing the chemical stability of a MOF by increasing the metal–ligand bond strength, a highly effective strategy to increase the stability of MOFs with labile metals.

On the other hand, the use of small metal cations confers stability against chemical degradation, as shown for several MOFs constructed with trivalent and tetravalent cations such as Al^3+^, In^3+^, Sc^3+^, and Cr^3+^ and Zr^4+^, V^4+^, and Ti^4+^, respectively. These metal centres tend to form robust clusters, increasing the stability of the framework towards corrosive gases. For example, MIL-101(Cr) and MIL-101(Cr)-4F(1%) formed by [Cr_3_(μ_3_-O)(O_2_CR)_6_] clusters, are stable to SO_2_ and H_2_S;^20,34^ Mg-CUK-1 with [Mg_3_(μ_3_-OH)]^5+^ clusters is stable to H_2_S;^68^ MIL-53(Al)-TDC formed by *trans*-corner-sharing [Al(μ_2_-OH)(*p*-BDC)] polyhedra is stable to H_2_S.^69^ Additionally, the MFM-300 family formed by infinite chains of [M_2_(μ_2_-OH)_2_(C_16_O_8_H_6_)] (M = Al^3+^, In^3+^, Sc^3+^, V^3+^), has demonstrated to be stable to SO_2_, H_2_S, NH_3_, and NO_2_.^37,70,110,111,125^ This family represents an excellent example of how the non-covalent interactions formed between the framework surface and the host molecules provide to the additional structural stability and adsorption reversibility in almost all the studied cases. The principal interaction involves the μ_2_-OH motif as hydrogen bond donor and the gas molecule as an acceptor, followed by guest–ligand and guest–guest interactions. This last aspect has a direct impact on the recyclability of the MOF, where most of the materials with good cyclability exhibit similar host–guest interactions such as hydrogen bonds (strong and weak), electrostatic interactions inside the pore framework, and metal–guest interactions. For example: MFM-170,^19^ MIL-101(Cr)-4F(1%),^20^ are stable even after 50 SO_2_ adsorption–desorption cycles while the following MOFs are stable for different numbers of SO_2_ cycles [Ni_8_(OH)_4_(H_2_O)_2_(BDP_X)_6_] (10 cycles),^45^ SIFSIX-1-Cu (4–6 cycles),^51^ and MFM-300(Sc) (10 cycles).^37*c*^ Furthermore, Mg-CUK-1,^68^ MIL-53(Al)-TDC,^69^ and MFM-300(Sc)^70^ are stable for at least five adsorption–desorption cycles of H_2_S while Mg_2_(dobpdc),^109^ Co_2_Cl_2_ BBTA and Co_2_Cl_2_ BTDD,^108^ MFM-300(Al) (50 cycles),^111^ and UiO-67.^112^ Finally, MFM-300(Al) is not affected even by five cycles of NO_2_.^124^

## 8. Conclusions and future perspectives

Air pollution due to the emissions of toxic gases is the single largest environmental health risk around the world since it is responsible for one in eight premature global deaths, reduction of the biodiversity, crop damages and acidification of soils and waters. Thus, the development of efficient technologies for the capture of toxic gases (*i.e.*, NO_*x*_, SO_2_, NH_3_ and H_2_S) from static and mobile sources is essential, in order to achieve a cleaner environment. MOFs are amongst the most promising candidates for the capture of these toxic gases since their sorption selectivity is directly tuneable as a function of the topology and chemical composition of the pores, which can optimise the interactions between MOFs and these toxic molecules, affording enhanced gas adsorption properties. Although the main drawback of MOFs is their vulnerability to these highly corrosive gases which can compromise their chemical stability, remarkable examples have demonstrated high chemical stability to NO_*x*_, SO_2_, NH_3_ and H_2_S.

Chemically stable MOFs for the capture of SO_2_ have provided promising advances in the field and the understanding of the role of different chemical functionalities, is the key for accomplishing superior SO_2_ captures. Coordinatively unsaturated metal sites (open metal sites) incorporated into chemical stable MOFs (*e.g.*, MFM-170 and MIL-101(Cr)-4F(1%)) demonstrated the relevance of the interaction of these open metal sites and SO_2_ molecules. The design of new functionalised MOF materials with coordinatively unsaturated metal sites signifies an interesting alternative to obtain superior SO_2_ captures and therefore, the challenge is to be able to also incorporate chemical stability to these MOFs. Thus, high SO_2_ uptakes are related to large BET surface area, pore size and the open metal sites incorporation into MOFs, while structural (chemical) stability is associated with the type of metal, and the interactions adsorbate–adsorbent inside the pore.

Hydroxo-functionalisation (μ-OH groups) in structural stable MOFs (*e.g.*, MFM-300(M)) have shown excellent capabilities for the capture and detection of SO_2_ with high chemical stability and excellent cyclability involving a remarkably facile regeneration. The key for all of such outstanding properties is the preferential interaction between the functional group (μ-OH) and SO_2_, in which the μ-OH groups binds SO_2_ molecules through the formation of O=S=O(*δ*^−^)⋯H(*δ*^+^)–O hydrogen bonds, reinforced by weak supramolecular interactions with C–H atoms from the aromatic rings of the framework. The chemical stability of the MFM-300(M) family relies on the strength of the coordination bonds between the oxygen atoms form the carboxylic ligand (biphenyl-3,3′,5,5′-tetracarboxylic acid) and the M^3+^ metal centres.

Defective sites *a.k.a.*, crystal irregularities, composition inhomogeneities or defects in MOFs have exhibited increased SO_2_ adsorption capacities, energies, and SO_2_/CO_2_ selectivity. For example, in nickel pyrazolate MOFs it was demonstrated that the pre-synthetic introduction of amino and hydroxyl functional groups on the organic linkers and the post-synthetic modifications, synergistically increase the SO_2_ capture capacity of these MOF materials.

Halogen functionalised MOF materials (*e.g.*, hexafluorosilicate (SiF_6_^2−^) incorporation in SIFSIX MOFs) showed highly efficient removal of SO_2_ from other gases, at a very low SO_2_ concentrations, and excellent SO_2_/CO_2_ and SO_2_/N_2_ selectivities. These properties were attributed to the strong electrostatic interactions between the SO_2_ molecules and the SiF_6_^2−^ anions (S^*δ*+^⋯F^*δ*−^), assisted by dipole–dipole interactions with the ligand (O^*δ*−^⋯H^*δ*+^).

The identification of MOFs capable of capturing H_2_S under industrially practical pressure-swing desorption conditions, symbolises a very promising application which is based on the chemical stability of these materials. For example, stable MOF materials constructed with μ-OH functional groups (*e.g.*, MIL-53(Al)-TDC) exhibited high and fully reversible H_2_S capture where the formation of hydrogen bonds between H_2_S and the μ-OH functional group (a relatively weak interaction) facilitates the H_2_S cyclability.

The chemical transformation of H_2_S within the pores of MOFs to produce, *in situ*, polysulphides is a new and exciting discovery in MFM-300(M) and SU-101 MOF materials. In addition to the promising application as novel MOF-lithium/sulphur batteries, the chemical investigation behind them, provided addition information on the electrochemical potential of MOFs which could guide new interesting properties to be discovered. Then, although the polysulphide formation was explained in terms of a “disproportionation” type reaction (protons of H_2_S play the role of an oxidant and the sulphide plays the role of a reducing agent), other hypotheses need to be explored. What if the polysulphide formation is a consequence of reversible metal–ligand bonding upon the adsorption of H_2_S? We anticipate new perspectives will be shortly investigated.

Paradoxically, the role of H_2_S as an endogenous biological mediator in the human body is crucial. For example, H_2_S executes a vital activity in the regulation of blood pressure, neurotransmission, anti-inflammatory mechanism, anti-oxidation, angiogenesis and apoptosis. Thus, the delivery and detection of H_2_S levels in living cells and organisms is an outstanding application for MOF materials. For example, Ni-CPO-27 showed a promising release of H_2_S (under physiological conditions) with a short induction period of approximately 5 min. In the field of H_2_S detection, for example, a post-synthetic modification of ZIF-90 with malononitrile (N≡C–CH_2_–C≡N), exhibited a specific reaction with H_2_S completing an enhancement of photoluminescence, which constitutes the base for the detection of H_2_S. Thus, the development of this research is expanding by the fundamental principle of introducing a reactive site for H_2_S within MOF material (monitored by fluorescence), which can work under physiological pH, to detect exogenous H_2_S in living cells, while the MOF-probe shows low toxicity and high biocompatibility.

Although most MOF materials have exhibited poor chemical stability towards NH_3_, chemically stable MOFs constructed with different chemical functionalities have shown interesting NH_3_ capture performances.

Coordinatively unsaturated metal sites incorporated, for example, in bisbenzenetriazolate and triazolate MOFs, showed high and reversible NH_3_ uptakes with strong interactions between the open metal sites and NH_3_. The chemical stability of these MOF materials was attributed to the strength of the N atom coordinated to divalent metal centres. Mg_2_(dobpdc) demonstrated high NH_3_ capture and chemical stability to not only dry NH_3_, but also to wet NH_3_. Interestingly, it was experimentally and computationally demonstrated that NH_3_ was preferentially adsorbed, by coordination bonds, to the coordinatively unsaturated metal sites of Mg_2_(dobpdc). The exceptional chemical stability of this Mg^2+^-based MOF material credited to the higher affinity of Mg^2+^ to oxygen atoms than nitrogen atoms, as confirmed by van der Waals (vdW)-corrected density functional theory (DFT) calculations.

The μ-OH functionalised MFM-300(M) family demonstrated remarkable NH_3_ adsorption properties, high chemical stability, high uptakes and attractive cyclabilities. Not surprisingly, as in the case of SO_2_ and H_2_S, the preferential adsorption sites of the MFM-300(M) materials were found at the hydroxo functional group. For example, in the case of MFM-300(Al) the adsorption of NH_3_ showed an atypical adsorption mechanism where adsorbent and adsorbate experienced a rapid site-exchange *via* reversible formation and cleavage of O–H and O–H_(from NH_3_)_ chemical bonds, in other words a pseudo-chemisorption binding mechanism.

As previously described in the SO_2_ capture section, crystal irregularities can provide very particular physical and chemical properties. Then, defects in MOF materials also demonstrated remarkable NH_3_ adsorption properties. For example, UiO-67 and UiO-bpydc containing 4,4′-biphenyl dicarboxylate and 4,4′-(2,2′-bipyridine) dicarboxylate ligands, despite their structural similarities demonstrated a drastic difference in the NH_3_ adsorption properties when the biphenyl groups in the organic ligand were replaced by the bipyridine moieties. Such replacement can confer flexibility to the framework in the context of mainly ligand “flipping” but without significant pore volume alteration.

Current investigations in NO_*x*_ capture have pointed out that chemical stable MOFs represent an attractive alternative for storage, selective separation, and/or catalytic transformation of NO_2_. For example, the synergistic effect between the organic ligand and the inorganic building block in MFM-300(Al) showed the stabilisation of highly reactive NO_2_ species within the micropores. The optimal uptake and selectivity of MFM-300(Al) for NO_2_ was attributed to the existence of both host–guest and guest–guest interactions. The former involves mainly the hydrogen bonding interaction between NO_*x*_ and the hydroxo functional group, while the latter refers to the supramolecular interactions between the guest molecules in their monomeric (NO_2_) and dimeric (N_2_O_4_) form.

Interestingly, the design of MOF materials for storage and controlled release of NO have gained relevance in biomedicine, as it has been shown that the exposure to controlled doses of NO induces wound healing and prevents the formation of blood clots. For example, CPO-27-Ni exhibited highly efficient adsorption, storage, and release cyclable profile for NO with a high stability to water, allowing a controlled release of NO when exposed to moisture.

Despite the significant progress in the capture of NO_*x*_, SO_2_, NH_3_ and H_2_S pollutants by MOF-based technology, further research in this field is required to overcome some of the main challenges: chemical stability, reusability, and suitable functionalisation. Considering the large number of MOF materials synthesised to date (over 70 000 structures reported in the CCD), it is crucial to understand what makes the above-reported MOFs so chemically stable. Thus, this perspective summarises their most important characteristics (metal centre, strength of the metal–ligand bond and functional groups) that should be further investigated in order to be able to design tailor-made MOFs for the capture of corrosive gases. Additionally, an important aspect to consider is the study of breakthrough experiments and selectivity calculations, where more realistic conditions for industrial applications are investigated, providing a direct performance comparison of MOFs to classic materials such as activated carbon, zeolites and metal oxides. On the other hand, the synthesis scalability of MOFs is one of the weak points to contemplate for corrosive gas adsorption applications, as only a few materials can be synthesised on an industrial scale. We desire that this perspective can provide useful information on the significant progress of this field and inspire new investigations to be carried out.

## Author contributions

E. M.-A., M. L. D.-R., and M. J. V.-H. conducted the literature research and drafted the manuscript. V. J. and I. A. I. conceived the topic and structure of the article. Outlined, drafted and supervised the completion of the manuscript. All authors reviewed and contributed to this manuscript.

## Conflicts of interest

There are no conflicts to declare.
